# The economics of primary prevention of cardiovascular disease – a systematic review of economic evaluations

**DOI:** 10.1186/1478-7547-5-5

**Published:** 2007-05-14

**Authors:** David LB Schwappach, Till A Boluarte, Marc Suhrcke

**Affiliations:** 1 Research Institute for Public Health and Addiction, Zurich, Switzerland; 2 Department of Health policy, University Witten-Herdecke, Witten, Germany; 3 WHO European Office for Investment for Health & Development, Venice, Italy

## Abstract

**Background:**

In the quest for public and private resources, prevention continues to face a difficult challenge in obtaining tangible public and political support. This may be partly because the economic evidence in favour of prevention is often said to be largely missing. The overall aim of this paper is to examine whether economic evidence in favour of prevention does exist, and if so, what its main characteristics, weaknesses and strengths are. We concentrate on the evidence regarding primary prevention that targets cardiovascular disease event or risk reduction.

**Methods:**

We conducted a systematic literature review of journal articles published during the period 1995–2005, based on a comprehensive key-word based search in generic and specialized electronic databases, accompanied by manual searches of expert databases. The search strategy consisted of combinations of freetext and keywords related to economic evaluation, cardiovascular diseases, and primary preventive interventions of risk assessment or modification.

**Results:**

A total of 195 studies fulfilled all of the relevant inclusion criteria. Overall, a significant amount of relevant economic evidence in favour of prevention does exist, despite important remaining gaps. The majority of studies were cost-effectiveness-analyses, expressing benefits as "life years gained", were conducted in a US or UK setting, assessed clinical prevention, mainly drugs targeted at lowering lipid levels, and referred to subjects aged 35–64 years old with at least one risk factor.

**Conclusion:**

First, this review has demonstrated the obvious lack of economic evaluations of broader health promotion interventions, when compared to clinical prevention. Second, the clear role for government to engage more actively in the economic evaluation of prevention has become very obvious, namely, to fill the gap left by private industry in terms of the evaluation of broader public health interventions and regarding clinical prevention, in light of the documented relationship between study funding and reporting of favourable results. Third, the value of greater adherence to established guidelines on economic evaluation cannot be emphasised enough. Finally, there appear to be certain methodological features in the practice of economic evaluations that might bias the choice between prevention and cure in favour of the latter.

## Background

In the quest for public and private resources, prevention continues to face a difficult challenge in obtaining tangible public and political support. In part this is because contrary to curative care, prevention has no identifiable beneficiaries and is commonly characterised by immediate costs and delayed benefits. In addition – and this has been the basic motivation for this study – the economic evidence in favour of prevention is said to be largely missing [1,2]. The purpose of this article is to examine the main characteristics, weaknesses and strengths of the existing evidence regarding economic evaluations of primary prevention, i.e., the prevention of disease before it occurs for the first time, including health promotion, screening for risk factors and risk factor modification through clinical prevention efforts. We concentrate on the evidence regarding primary prevention targeting cardiovascular disease (CVD) event or risk reduction. CVD accounts for the largest share of mortality in most high-income countries, and increasingly also in developing countries [3]. By focusing on CVD prevention we are also able to capture a set of the risk factors that account for the largest share of the disease burden in Europe, such as tobacco consumption, high blood pressure, high body mass index, and physical inactivity.

Based on empirical analyses of the published literature, we describe and summarize the quantity, content and type of existing health economic evidence in relation to diseases, interventions and health care systems that has been generated so far. These results should also provide indications as for areas in which evidence is particularly scarce or even absent. To allow for both a comprehensive overview and more in depth analysis, the review is divided in two parts:

1) In a first step, we identified all full economic analyses that evaluated primary prevention activities aiming at reducing the burden of cardiovascular diseases. These evaluations were assessed in terms of various key parameters that allow us to illustrate and quantify the published evidence, to describe interventions and preventive strategies that have been intensively evaluated, and finally to draw conclusions on areas that have been outside researchers' focus so far. This part of the study thus generates data about the basic characteristics of all identified economic evaluations conducted in the area of primary prevention of cardiovascular disease.

2) In the second step, we concentrate on a subsample of studies to conduct an in-depth analysis of economic evaluations of interventions targeted at specific risk factors for cardiovascular diseases. We selected dietary intake, weight management and physical activity as major *targets *for risk reduction. This focus has been set because these are the main proximate drivers of obesity, no doubt a key public health challenge that has been less researched than, for instance, smoking and excessive alcohol consumption. We did not select any particular type of primary preventive interventions, so that this second part of the study should depict the available evidence on the entire continuum of primary prevention: public health policy, health promotion, and clinical prevention. While the subsample is certainly not representative of all economic evaluations assessed in phase 1 of our study, we expected to detect certain methodological and systematic problems and key questions that may be relevant to the majority of evaluations of primary prevention activities, irrespective of the particular intervention under study.

## Methods

### Literature search and study selection

We searched the databases Embase, Pubmed (Medline), NHS-Pharmline, NHS EED, and OHE HEED for relevant articles. In addition, the Harvard cost-effectiveness registry was manually searched. The searches were conducted in May 2006. We also consulted a database of German economic evaluations published in 1990–2004 that was the result of a prior study focusing on the German health care system [4]. The search strategy consisted of combinations of freetext and MeSH ("Medical subject heading") terms related to economic evaluation, cardiovascular diseases, and primary prevention interventions for risk assessment or modification (see Appendix 1). "Wild cards" and abbreviations of terms were used. The retrieved records were further refined for the relevant year range and, where available, limited to journal articles. Studies were included in the review when they fulfilled all of the inclusion criteria listed in Table 1.

**Table 1:** Inclusion criteria

**Contentual features:**
■ Studies evaluating primary prevention activities targeted at cardiovascular event or risk reduction, i.e., prevention of the disease before it occurs for the first time;
■ Objective: Screening for and modification of risk factors for primary cardiovascular events;
■ Population: Persons at increased risk but without evidence of cardiovascular disease;
■ Endpoints: Cardiovascular outcomes/events or modified risk factor;
**Formal features:**
■ Full economic evaluation, i.e., comparative analysis of costs and outcomes of at least two alternatives;
■ Applied study (trial generating primary data or modelling of secondary data). Methodological and general articles, letters and abstracts were excluded;
■ Assessment of, or application to the US, Canadian or European health care systems;
■ Journal articles, i.e., exclusion of books, HTA reports, grey literature;
■ Published between 1995–2005.

### Data extraction and critical appraisal

The identified articles were retrieved as fulltexts. We developed a checklist comparable to that used by other researchers to extract data alongside review of the original studies. The following data were extracted from all included economic evaluations (study phase 1):

- the publication language

- the country of origin/investigated health care system

- the study design (randomized clinical trial, observational study, modelling, or combination of trial and modelling)

- the type of economic evaluation (cost-effectiveness analysis, cost-utility analysis, cost-benefit analysis, cost-minimization analysis, cost-consequence analysis)

- the intervention target (targeted risk factors)

- the target population in terms of age, gender, risk factors

- the type of primary prevention intervention (health promotion, i.e., addressing a community of people; screening, i.e., identification persons at higher risk for a disease; clinical prevention, i.e., individually delivered to a certain patient)

- the benefit measures: natural units (extracted in detail), life years gained, QALYs (DALYs), Willingness to pay (benefit in monetary terms)

- the study perspective (as stated by authors)

- the funding of the study (as stated by authors)

The data extraction form did *not *include explicit quality ratings. For all studies, we used only the information provided in the original publication. Each paper was independently read by a single, trained researcher. After critical appraisal, information collected in data extraction forms were transferred to an electronic database. Based on this initial step of the systematic review, we identified the subcollection of evaluations that assessed the economics of primary prevention interventions targeted at dietary intake, weight management, or physical activity. These studies were the objective of our in-depth analyses. Each paper in the subsample was independently read by two trained researchers. In addition to those criteria already extracted in phase 1, we closely followed guidelines on economic evaluations and selected additional key aspects according to which the study-specific information was disaggregated [5,6].

These included:

- The specific intervention under study

- the comparator against which the interventions was evaluated

- the target group

- the time horizon

- the kind of discounting (rate and base year)

- the cost components included, the incremental cost-effectiveness ratio (ICER)

- the methods employed to handle uncertainty, and

- the consideration of future costs.

For each of the studies included in phase 2, case reports were written that summarized the basic objective, methodology, and results of the evaluations as well as underlying key assumptions.

### Data analysis

Descriptive statistics were used to analyze the main characteristics of the included economic evaluations and to examine associations between extracted categories, e.g., type of intervention and target group. To assess potential developments over time, we split the overall period covered into 1995–2000 and 2001–2005. Comparisons were made using chi-square tests. A p-value of less than 0.05 was considered significant. All analyses were performed using STATA 9 software [7].

## Results

**Figure 1:**
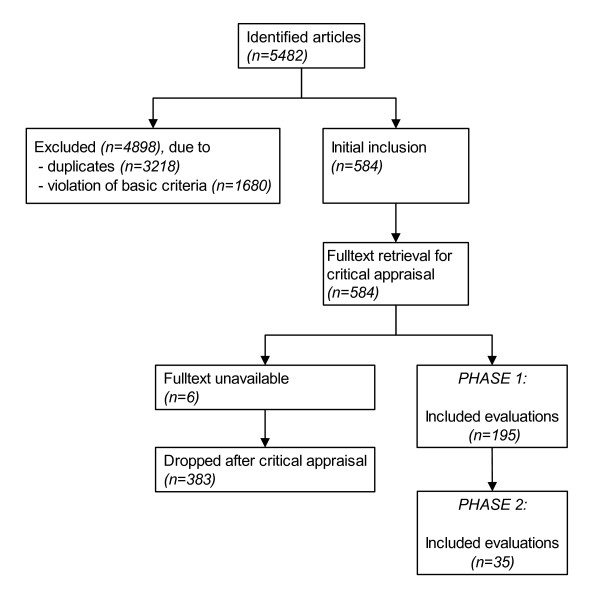
Overview of in- and exclusion of studies.

The systematic literature search initially identified 5,482 candidate articles, of which 584 were selected for fulltext retrieval (figure 1). The majority of articles were discarded at this initial stage mainly because they were duplicates (n = 3,218), or it was obvious from the bibliographic data and abstract that they violated basic inclusion criteria (e.g., abstracts in congress supplements) (n = 1,680). Of the 584 fulltexts selected for retrieval, a further 389 were dropped after the critical appraisal because they failed one or more inclusion criteria. Most studies were excluded because they were not applied studies or not full evaluations.

### Studies analyzed in phase 1 of the review

In summary, 195 articles were included in phase 1 of this systematic review. The majority of the studies included were published in English language (95%) and concerned the health care systems of the United Kingdom (21%) or the USA (37%). Half of the studies (45%) were published during 1995–2000 compared to 107 studies (55%) published in the 2001–2005 period. In what follows we describe and briefly discuss the main characteristics of the studies included.

#### Category of primary prevention

Looking at the characteristics of the studies concerning the prevention category they are evaluating, it is obvious that the vast majority of studies (87%) evaluated measures of clinical prevention, and pharmacotherapy in particular (56%) (Table 2). Only a minority of the studies (10%) examined health promotion activities addressing the health behaviour of communities of people including education, advertising, or legislation. Screening for risk factors for cardiovascular disease was only assessed in a small fraction of studies (3%), all published between 2001–2005.

**Table 2:** Interventions by prevention category

	Overall (n = 195) No. (%) of studies	% of studies within category
**Health Promotion**	**20 (10)**	**100**
Education	7 (4)	35
Advertising	4 (2)	20
Legislation	8 (4)	40
Other Health Promotion*	1 (1)	5
**Screening**	**5 (3)**	**100**
**Clinical Prevention**	**170 (87)**	**100**
Health Education	35 (18)	21
Pharmacotherapy	110 (56)	65
Surgery	1 (0.4)	1
Practitioner Education	13 (7)	8
Screening and Clinical Intervention	11 (6)	7

* Summarized over extracted categories

#### Intervention targets

Analyzing the studies included in this review in terms of the intervention targets, i.e., the risk factors, they examined, 33% of the 195 evaluations addressed dyslipidemia, 21% targeted smoking and 13% concerned high blood pressure (Table 3). Only small fractions of the published studies evaluated interventions targeted at high blood glucose levels (7%), obesity (5%) or dietary intake (6%). There were significant differences in the targeted risk factors between study settings: North American studies were more likely to evaluate the economics of interventions addressing smoking, dietary intake and dyslipidemia, while studies assessing interventions within a European context more often addressed high blood pressure and glucose levels.

**Table 3:** Intervention targets by study setting

	North America No. (%) of studies	Europe No. (%) of studies	Total No. (%) of studies
Intervention target evaluated			
Smoking	24 (28)	16 (16)	40 (22)
Obesity	5 (6)	5 (5)	10 (6)
Physical inactivity	2 (2)	3 (3)	5 (3)
Dietary intake	8 (10)	4 (5)	12 (7)
Dyslipidemia	30 (35)	26 (27)	56 (31)
High blood pressure	6 (7)	18 (18)	24 (13)
High blood glucose levels	3 (4)	10 (10)	13 (7)
Atrial fibrillation	1 (1)	2 (2)	3 (2)
Various	6 (7)	14 (14)	20 (11)

#### Age of target population

**Figure 2:**
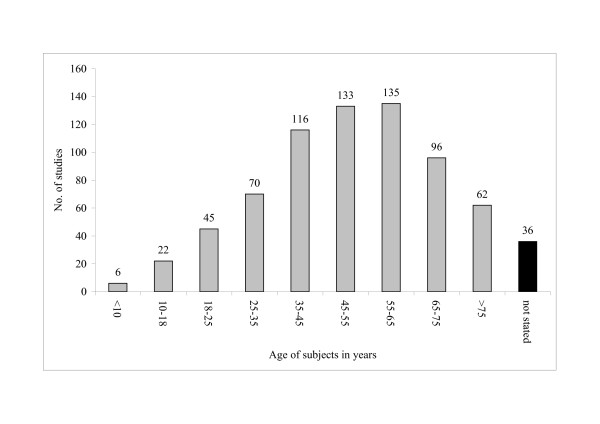
Age of target population in included studies, as stated by authors.

We used the data presented in the original publications to extract either age-related inclusion criteria or descriptions of the study sample. Due to this generalized post-hoc classification, evaluations may cover multiple age groups (e.g. "25 to 34" and "35 to 44"). Unfortunately, a significant number of evaluations (19%) did not clearly document the age of patients included in their study. As can be seen from the distribution of covered or targeted age groups (figure 2), the vast majority of studies included subjects aged 35–64 years in their studies. Many evaluations assessed health care delivered to seniors: The health of persons aged 65–74 and 75 and above was addressed in 60% and 39% of studies respectively. Contrary, only very few studies evaluate interventions targeted at children (0 to 18 years) or young adults (18 to 35 years).

#### Economic evaluation methods

Of the reviewed studies, the vast majority were cost-effectiveness-analyses that had used "life years gained" (37%) or clinical outcome measures (e.g., number of avoided CVD incidents) as benefit measure (27%) (Table 4). A relatively high number of publications (11%) reported no "true" economic evaluations but observational studies with information on costs that left health outcomes disaggregated and used no summary measure of benefit (cost-consequence analyses). Comparing changes in the chosen analytic frameworks over time, a systematic trend towards more comprehensive methods can be seen. The number of cost-utility analyses increased significantly from 6 published in the period from 1995–2000 (7%) to 32 studies (30%) published during 2001–2005 (p = <.001). During the same period there was a slight decrease of cost-effectiveness studies using "life years gained" as measure of benefit (47% in 1995–2000 vs. 30% in 2001–2005, p = .017).

**Table 4:** Characteristics of economic evaluations by publication period

	1995–2000 (n = 88) No. (%) of studies	2001–2005 (n = 107) No. (%) of studies	1995–2005 (n = 195) No. (%) of studies
By Study Type			
Cost effectiveness (life years gained)	41 (47)	32 (30)	73 (37)
Cost effectiveness (clinical outcome)	25 (28)	28 (26)	53 (27)
Cost utility	6 (7)	32 (30)	38 (20)
Cost consequence	11 (13)	10 (9)	21 (11)
Cost minimization	4 (5)	3 (3)	7 (4)
Cost benefit	1 (1)	2 (2)	3 (2)
By Perspective			
Societal	15 (17)	23 (22)	38 (20)
Third Party Payer	28 (32)	38 (36)	66 (34)
Health care provider	1 (1)	3 (3)	4 (2)
Patient	1 (1)	1 (1)	2 (1)
Other*	3 (3)	1 (1)	4 (2)
Not stated	40 (46)	41 (38)	81 (42)
By Funding source			
Government	20 (23)	31 (29)	51 (26)
Industry	22 (25)	29 (27)	51 (26)
Foundation	10 (11)	5 (5)	15 (8)
Other*	0 (0)	7 (7)	7 (4)
Not stated	36 (41)	35 (33)	71 (36)

* Summarized over extracted categories

#### Study perspective

The perspective adopted in an economic evaluation can heavily influence its results and clear reporting of perspective is therefore crucial. However, in only 59% of the studies included in the main sample, the authors explicitly documented the perspective the study was undertaken from. This fraction increased only slightly over time and did not differ significantly among study settings. The majority of evaluations reporting study perspective were undertaken from the viewpoint of a third party payer (34%). Among the studies that claimed to be undertaken from the more comprehensive societal point of view, 13 were cost-utility analyses and 18 cost-effectiveness analyses with "life years gained" as benefit measure.

#### Funding

Only 64% of evaluations provided provided statements on funding. Among those, financial support by the industry (26%) and by government (26%) were most frequent (Table 4). Sponsorship was strongly associated with the intervention type: Among the studies that reported funding by the industry, 89% evaluated pharmaceutical interventions. By contrast, 86% of those studies that disclosed funding information and evaluated health promotion activities received governmental grants. The source of funding was also related to measures of benefit and documented study perspective in the reviewed studies: evaluations that reported governmental financial support were more likely to express interventions' outcomes in terms of more comprehensive benefit measures (QALYs, DALYs, "life years gained", or "willingness to pay") (77%), while industry sponsored studies used comprehensive measures in only 47% of cases and were more likely to express benefits in terms of fragmented natural units (53%). Industry-funded evaluations that documented study perspective were significantly less likely to adopt a societal perspective as compared to studies financed by government (12% vs. 53%, p = <.001).

### Studies analyzed in phase 2 of the review

Of the 195 articles included in phase 1, 35 studies (18%) evaluated interventions targeted at dietary intake, weight management, or physical activity, either exclusively or in a multifaceted approach with other risk factors, and thus built the sub-sample for the more in depth phase 2 of this review. Of the 35 studies, 17 (49%) evaluated prevention in a US or Canadian setting, and 17 studies (49%) were related to European health care systems. One study used a multinational approach.

Looking at the type of intervention evaluated in the studies, 74% (n = 26) assessed clinical prevention efforts and 26% (n = 9) evaluated health promotion activities. Evaluated interventions most commonly addressed dietary intake (n = 12), obesity (n = 10) or various risk factors (n = 7). The time horizons chosen for the evaluations showed a variety of 13 alternatives ranging from as short as "4 month" to a "lifetime" framework. Concerning the methods utilized to handle uncertainty in parameter estimates, 29% (n = 10) did not document use of sensitivity analysis, 20% (n = 7) used one-way and 40% (n = 14) multi-way analysis sensitivity. 11% (n = 4) employed probabilistic analysis. Out of the subsample of phase 2, only 13 studies reported incremental cost-effectiveness ratios.

While the original ambition may have been to compare the studies included in the sub-sample in order to propose a ranking of "best buys" in primary prevention, the diversity in the methods and data used to evaluate interventions prevents literal comparison of the resulting cost-effectiveness ratios. (Nevertheless, to provide a resource for other researchers and interested parties, the detailed tabulation of the data extracted from the 35 studies in Appendix 2 includes these criteria.) The use of comprehensive benefit measures is generally far better suited to enable a comparison of different interventions. Unfortunately, only 20 of the studies included in our phase 2 subsample (57%) used at least one comprehensive benefit measurement, such as QALYs, DALYs, "life years gained", or a monetary value – reflecting the findings obtained for the larger and less-restricted sample in phase 1. The remainder of the studies used specific clinical parameters as outcome measures (43%), such as the change in blood lipid levels, weight loss or behavioural change, making it almost impossible to weigh studies against each other or even to estimate the relevance of their associated benefits. Besides this "unit" difference, other differences in the methods lead to problems in comparing the studies among each other. Below we elaborate on just two of them – the type of costs to include and the discounting procedure.

#### Type of costs

The studies included in our review show large variability in the type of costs included. 34 out of 35 studies analyzed in the subsample included "direct" costs into their calculation. Only 9 studies also included "productivity losses" (often termed "indirect costs") in their assessment of costs. The vast majority of these studies used the "human capital approach" to value productivity losses. 43% of the studies (n = 15) also incorporated future cost savings into their evaluation, while only 9% (n = 3) did include future costs caused by the additional consumption of medical goods due to enhanced life expectancy. Obviously, such diversity seriously impedes any direct comparison of the studies included, all the more so because the pricing of units of resources (e.g., doctor consultations) also varies with the chosen perspective of the analysis (e.g., patient, society, etc.).

#### Discounting

The way in which future costs and benefits of an intervention are being discounted, i.e., adjusted for different timing of costs and benefits and transformed to their 'present value', greatly influences the economic evaluation of a given intervention [8]. The higher the discount rate or the further ahead in the future the cost/benefit, the lower the net present value of the monetary figures. A glance at Table 5 confirms that the 35 studies assessed in phase 2 have used very different approaches to discounting: Nearly every second study (43%) did not apply any discounting to their results. Of those studies that inflated both costs and benefits (46%), the vast majority used identical discount rates for both all costs and all benefits. A discount rate of 3% was most common both for costs and benefits. As mentioned above, in one study the discount rate differed even by the type of intervention evaluated: the benefits of pharmacotherapy with statins were discounted at a rate of 6% (to take into account side-effects), while the effects of diet were discounted at a rate of only 3%.

**Table 5:** Methodology for discounting in studies (phase 2)

	No. (%) of studies (n = 35)
Discounting approach	
No discounting	15 (43)
Identical discount rates for costs and benefits	14 (40)
Different discount rates for costs and benefits	2 (6)
Only discounting of costs	2 (6)
Only discounting of benefits	2 (6)
Discount rate for costs	
0%	17 (49)
3%	11 (31)
5%	5 (14)
6%	2 (6)
Discount rate for benefits	
0%	17 (49)
1.5%	1 (3)
3%	11 (31)
5%	5 (14)
6%	1 (3)

## Conclusion

The purpose of this article has been to examine the main characteristics of the existing evidence regarding economic evaluations of primary prevention, here specifically applied to CVD event and risk reduction. The broader policy relevance of this issue is obvious: the allocation of public and private resources overwhelmingly favours curative interventions as opposed to prevention. This is for instance illustrated by the 3.2% share out of government health expenditures that went into the category 'prevention and public health' in the 19 OECD countries for which recent data was available [9]. Taking this data literally (which is problematic given the challenge of measuring expenditures on prevention), it is tempting to infer that these spending levels reflect a sub-optimal level of prevention, from a social welfare perspective [10]. Assuming this is the case, we hypothesised that a lack of evidence demonstrating "returns on investment" in prevention may have contributed to the low priority assigned to it. Our results suggest that a significant amount of economic evidence in favour of prevention does exist. However, at the same time there are important gaps in the published literature that call for more research.

The majority of the 194 reviewed studies were cost-effectiveness analyses that express benefits as "life years gained", were conducted in a US or UK setting, assessed clinical prevention (mainly drugs targeted at lowering lipid levels), and referred to subjects aged 35–64 years old with at least one risk factor. The main gaps or limitations in the reviewed studies, and of the review method per se, were:

- Only very few studies assessed broader health promotion interventions targeted at obesity, physical inactivity or dietary intake in children or young adults.

- Interventions targeting children or young people have only very rarely been evaluated in economic terms, despite the high expected benefits that is generally attributed to "early" prevention.

- The comparability of results between studies is severely limited by the marked differences in the methodologies and definitions applied.

The relative lack of broader health promotion evidence we report does not imply that there was no economic evidence in its favour, if we take the reviewed studies literally. One relatively strong area of evidence relates to smoking prevention, in particular to smoking cessation and taxation- issues that have deliberately not been analysed in depth here, because they have been addressed elsewhere [11,12]. To quote but one study from our sample in Phase I, Wang et al. [13] demonstrate that a project to prevent tobacco use among school children has proved to be even cost-saving. Importantly, this is also one of the very few studies explicitly targeting adolescents.

Compared to smoking – a comparatively "old" challenge – it is not surprising that thus far significantly less (cost-)effectiveness evidence has been accumulated in terms of the prevention of dietary intake and obesity. The most comprehensive study – when judged by the set of interventions covered – demonstrated remarkably favourable cost-effectiveness evidence for a number of such interventions, e.g., legislation to reduce salt content in processed food or broad-based health education [14]. A further advantage of the same study was that it allowed for an assessment of various combinations of personal, preventive and curative, as well as non-personal interventions [14]. This brings the situation analysed much closer to that faced by public health and health care decision makers, where the choice is typically not between one intervention and another, but rather between sets of interventions.

Despite these important contributions, the evidenced bias on curative, clinical care is in line with the findings of other studies. Pritchard had earlier examined the characteristics of all economic evaluations included in the HEED database [15], concluding that only 10% of *all *evaluations assessed preventive care. The scarcity of broader public health interventions in the present review as well as in other studies may be explained by at least three factors. (1) The applied formal analytical technique has strong requirements that may be less amenable to assessing "broader" types of interventions. (2) Economic evaluations are often conducted as a necessary condition for coverage by statutory health insurances, and therefore concentrate on interventions that are – in principle – subject to coverage, such as pharmaceuticals. Factors that contribute to a healthy living but are traditionally assigned to individual, private life, such as healthy nutrition, often lack this "pressure to prove" since there is no administrative institution deciding upon coverage. (3) The fact that the majority of studies have been financed by the private industry may in part explain the overrepresentation of pharmaceutical interventions.

As others, we observed a very high level of variability in the methods utilized in economic evaluations [4,16,17]. We did not formally address methodological quality or adherence to established guidelines, and there may be good reasons for different study designs for different purposes that lack comparability but are all, each by themselves, legitimate and in concordance with best practice recommendations. In addition, differing methodological approaches do not per se limit comparability as long as methods are clearly documented and results are presented in a way that would enable conversion to a common unit. However, we encountered severe limitations in transparency and documentation of main study details, e.g., chosen study perspective. In addition, significant numbers of studies failed to provide details on units of resource consumption, costing year, currencies and other details, as required by virtually all scientific guidelines on economic evaluation.

The reviewed evidence as such offers little to settle the debate on the relative benefits of prevention versus cure. However, there appear to be certain methodological features in the current practice of economic evaluations that might bias the choice between prevention and cure in favour of the latter. In this article we have discussed in particular the issue of the treatment of future costs and the discounting of future health benefits.

The diversity in the discounting procedures applied in the reviewed studies does not facilitate the comparison between the different interventions proposed, and raises the question whether there exists one "correct" way of discounting costs and benefits. As there is general agreement on the need for the discounting of costs, the zero discounting rate applied in 15 studies in our sample is deeply concerning. Still, the scientific debate about how to correctly treat future health benefits remains unresolved [18]. The debate revolves around several aspects, including the issue of time-varying discount rates to reflect time preference [19,20] or discounting models in relation to effectiveness measures [21], but the core controversy centres mainly on whether the health benefits of a given intervention should be discounted at a lower rate than the costs or at the same, uniform rate [22,23].

Whatever the "correct" way of discounting is – a question we cannot address properly here -the current predominant practice of uniform discounting does have implications for the choice between prevention versus cure at large. Since in the case of prevention health benefits tend to occur further ahead in the future compared to treatment, uniform discounting leads to the prioritization of immediate treatment at the expense of prevention. Differential discounting, with discount rates for health benefits lower than for costs, would certainly devalue future health benefits by much less than uniform discounting does. As a result, prevention would appear more favourable than it presently seems in economic evaluations. (Clearly, also the level of the uniform discount rate per se critically affects the choice between prevention and cure.) Similarly, the introduction of variable (but uniform) discount rates over time could lead to a greater appreciation of the benefits of prevention. [24-27].

Similar conclusions can be drawn from our findings relating to the inclusion of future costs: we found a high level of variability in the extent to which future costs are included in economic evaluations which is not suprising since guidelines rarely provide clear recommendations [28]. The question which costs to consider in the assessment of interventions has important and systematic consequences for their demonstrated favourability, and, as such, a high potential for introducing bias towards specific areas of care [29-32]. The in- or exclusion of future costs and savings due to related illness *and *"unrelated" illnesses is of particular relevance for the evaluation of prevention. Van Baal et al. exemplified the effects of including future costs of unrelated illnesses by computing different cost-utility ratios for smoking cessation interventions in different age groups [33]. Including health care costs of unrelated medical care in life years gained increased ratios, but excluding unrelated medical costs favoured smoking cessation interventions targeted at older smokers over those at younger smokers. They conclude that for primary prevention only a cost utility ratio that includes both the costs and effects of unrelated medical care meets the criterion of internal consistency. From a pro-preventive perspective, the common practice of excluding future costs may bias cost-effectiveness analyses against such interventions, and, within such interventions, against younger age groups.

In interpreting the above findings, the limitations of our study need to be borne in mind [34]:

(1) We limited our search to specific databases, year ranges and publication media and a number of tight inclusion criteria were pre-specified. Thus we cannot rule out having overlooked important evidence, for instance from the grey literature.

(2) We did not restrict our sample to single countries or small geographic domains but included studies applied to North American and European countries. While this approach is valuable in describing the available evidence from a meta perspective, it limits the use of our results for health policy and decision making due to a lack of generalizability of health economic data across countries [35].

(3) As is characteristic of systematic reviews in general, we were only able to identify, review and summarize *what has been published*, instead of *what has not been published*. The fact that studies that do not prove what has been expected by researchers (or funding parties) are less likely to get published introduces systematic bias, known as "publication bias". Hence, studies that report favourable results are overrepresented and there is a tendency to overestimate efficiency and effectiveness of health care interventions. The phenomenon of "publication" bias has been well described for the biomedical literature and has been identified as a major threat to reliable syntheses of outcomes research [36-40]. Economic analyses is even more vulnerable to publication bias [41].

The relative scarcity of economic evaluations of broader health promotion interventions has implications far beyond the merely scientific ones: in a situation that has essentially all high-income countries and increasingly also the developing world grappling with the mounting challenge of obesity and other "lifestyle-related diseases". There is reason to doubt as to whether clinical intervention will be the most effective (or even cost-effective) way to tackle challenges of this kind. Yet, as public budgets are tightening as the result of demographic and technological change, proving value for money arguably is becoming ever more important to justify investment at the policy level.

How can the existing evidence gaps be closed? Above all, there is a strong efficiency case for an increased public role in the promotion and production of evidence on primary prevention [10]. First, this would serve filling the gap left by private industry in terms of the evaluation of broader public health interventions and health promotion. In addition, it could contribute to a less biased representation of the clinical prevention results, given the documented relationship between study funding and reporting of favourable results [42-46]. Given the diversity of approaches witnessed even within the smaller sample of studies analysed, a key component in the development of "better", i.e., more comparable evaluations should be a greater adherence to established guidelines on economic evaluation including, above all, a higher level of transparency in the applied methods.

## Competing interests

The author(s) declare that they have no competing interests.

## Authors' contributions

DLBS, TAB and MS conceived of the study and participated in its design. MS coordinated the study group. DLBS and TAB conducted the literature review. TAB coordinated data retrieval and extraction. DLBS performed the statistical analysis. All authors drafted the manuscript, and read and approved the final manuscript.

## Supplementary Material

Additional File 1 Appendix 1 - Search strategy Click here for file

Additional File 2 Appendix 2 - Details of studies included in phase 2 assessment Click here for file
