# Supplementary material for: The economics of primary prevention of cardiovascular disease – a systematic review of economic evaluations
Source: Cost Eff Resour Alloc. 2007 May 14;5:5. doi: 10.1186/1478-7547-5-5 (PMC1876202; doi:10.1186/1478-7547-5-5)
Supplement: Additional File 1 — Appendix 1 - Search strategy [file 1478-7547-5-5-S1.pdf]

Additional files

Appendix 1 – Search strategy

EMBASE

```
#1  EM74
#2  (((((((CT D "HYPERCHOLESTEROLEMIA" OR UT="HYPERCHOLESTEROLEMIA" OR
    IT="HYPERCHOLESTEROLEMIA" OR SH="HYPERCHOLESTEROLEMIA") OR (CT D
    "DYSLIPIDEMIA" OR UT="DYSLIPIDEMIA" OR IT="DYSLIPIDEMIA" OR
    SH="DYSLIPIDEMIA")) OR (CT D "HYPERTENSION" OR UT="HYPERTENSION" OR
    IT="HYPERTENSION" OR SH="HYPERTENSION")) OR (CT D "ISCHEMIC HEART
    DISEASE" OR UT="ISCHEMIC HEART DISEASE" OR IT="ISCHEMIC HEART DISEASE"
    OR SH="ISCHEMIC HEART DISEASE")) OR (CT D "CARDIOVASCULAR DISEASE" OR
    UT="CARDIOVASCULAR DISEASE" OR IT="CARDIOVASCULAR DISEASE" OR
    SH="CARDIOVASCULAR DISEASE")) OR (CT D "ISCHEMIC HEART DISEASE" OR
    UT="ISCHEMIC HEART DISEASE" OR IT="ISCHEMIC HEART DISEASE" OR
    SH="ISCHEMIC HEART DISEASE")) OR (CT D "CORONARY ARTERY DISEASE" OR
    UT="CORONARY ARTERY DISEASE" OR IT="CORONARY ARTERY DISEASE" OR
    SH="CORONARY ARTERY DISEASE")) OR (CT D "CORONARY RISK" OR UT="CORONARY
    RISK" OR IT="CORONARY RISK" OR SH="CORONARY RISK")) OR (CT D
    "CARDIOVASCULAR RISK" OR UT="CARDIOVASCULAR RISK" OR IT="CARDIOVASCULAR
    RISK" OR SH="CARDIOVASCULAR RISK")
#3  ((((((((CT D "PRIMARY PREVENTION " OR UT="PRIMARY PREVENTION " OR
    IT="PRIMARY PREVENTION " OR SH="PRIMARY PREVENTION ") OR (CT D
    "PREVENTIVE HEALTH SERVICE" OR UT="PREVENTIVE HEALTH SERVICE" OR
    IT="PREVENTIVE HEALTH SERVICE" OR SH="PREVENTIVE HEALTH SERVICE")) OR
    (CT D "PREVENTIVE TREATMENT" OR UT="PREVENTIVE TREATMENT" OR
    IT="PREVENTIVE TREATMENT" OR SH="PREVENTIVE TREATMENT")) OR (CT D
    "PREVENTIVE THERAPY" OR UT="PREVENTIVE THERAPY" OR IT="PREVENTIVE
    THERAPY" OR SH="PREVENTIVE THERAPY")) OR (CT D "PREVENTIVE HEALTH CARE"
    OR UT="PREVENTIVE HEALTH CARE" OR IT="PREVENTIVE HEALTH CARE" OR
    SH="PREVENTIVE HEALTH CARE")) OR (CT D "PREVENTIVE SERVICE" OR
    UT="PREVENTIVE SERVICE" OR IT="PREVENTIVE SERVICE" OR SH="PREVENTIVE
    SERVICE")) OR (CT D "PREVENTION" OR UT="PREVENTION" OR IT="PREVENTION"
    OR SH="PREVENTION")) OR (CT D "HEALTH EDUCATION" OR UT="HEALTH
    EDUCATION" OR IT="HEALTH EDUCATION" OR SH="HEALTH EDUCATION")) OR (CT D
    "PATIENT EDUCATION" OR UT="PATIENT EDUCATION" OR IT="PATIENT EDUCATION"
    OR SH="PATIENT EDUCATION")) OR (CT D "Health Promotion" OR UT="Health
    Promotion" OR IT="Health Promotion" OR SH="Health Promotion")
#4  (CT D "PROPHYLAXIS" OR UT="PROPHYLAXIS" OR IT="PROPHYLAXIS" OR
    SH="PROPHYLAXIS") OR (CT D "Screening" OR UT="Screening" OR
    IT="Screening" OR SH="Screening")
#5  3 OR 4
#6  2 AND 5
#7  (((CT D "SMOKING CESSATION" OR UT="SMOKING CESSATION" OR IT="SMOKING
    CESSATION" OR SH="SMOKING CESSATION") OR (CT D "Physical EXERCISE" OR
    UT="Physical EXERCISE" OR IT="Physical EXERCISE" OR SH="Physical
    EXERCISE")) OR (CT D "BODY WEIGHT" OR UT="BODY WEIGHT" OR IT="BODY
    WEIGHT" OR SH="BODY WEIGHT")) OR (CT D "WEIGHT REDUCTION" OR UT="WEIGHT
    REDUCTION" OR IT="WEIGHT REDUCTION" OR SH="WEIGHT REDUCTION")
#8  6 OR 7
#9  ((((((CT D "COST EFFECTIVENESS" OR UT="COST EFFECTIVENESS" OR IT="COST
    EFFECTIVENESS" OR SH="COST EFFECTIVENESS") OR (CT D "COST BENEFIT" OR
    UT="COST BENEFIT" OR IT="COST BENEFIT" OR SH="COST BENEFIT")) OR (CT D
    "COST MINIMIZATION" OR UT="COST MINIMIZATION" OR IT="COST MINIMIZATION"
    OR SH="COST MINIMIZATION")) OR (CT D "COST UTILITY" OR UT="COST UTILITY"
    OR IT="COST UTILITY" OR SH="COST UTILITY")) OR (CT D "ECONOMIC
    EVALUATION" OR UT="ECONOMIC EVALUATION" OR IT="ECONOMIC EVALUATION" OR
    SH="ECONOMIC EVALUATION")) OR (CT D "COST CONSEQUENCE" OR UT="COST
    CONSEQUENCE" OR IT="COST CONSEQUENCE" OR SH="COST CONSEQUENCE")) OR (CT
```

```
    D "Quality adjusted life year" OR UT="Quality adjusted life year" OR
    IT="Quality adjusted life year" OR SH="Quality adjusted life year")
#10 8 AND 9
#11 (CT D "article" OR UT="article" OR IT="article" OR SH="article") AND
    PY=1995 to 2005 AND PPS=Mensch
#12 10 AND 11
```

PUBMED (Medline)

```
      ("cost-benefit analysis"[MeSH] OR "Quality-Adjusted Life Years"[MeSH])
      AND ("Myocardial Ischemia /prevention and control"[MeSH] OR
      "Dyslipidemias/prevention and control"[MeSH] OR "Cardiovascular
      Diseases/prevention and control"[MeSH] OR "Smoking/prevention and
      control"[MeSH] OR "Myocardial Ischemia/economics"[MeSH] OR
      "Dyslipidemias/economics"[MeSH] OR "Smoking Cessation/economics"[MeSH]
      OR "Smoking/economics"[MeSH] OR "Cardiovascular
      Diseases/economics"[MeSH] OR "Obesity"[MeSH] OR "Nutrition"[MeSH] OR
      "Exercise"[MeSH])
AND   ("1995"[PDAT] : "2005"[PDAT])
NOT   (Letter[ptyp] OR Editorial[ptyp] OR review[ptyp])
```

NHS-Pharmline (Advanced search)

```
#1    Freetext: ("Costs" OR "QALY" OR "quality-adjusted life year" OR "Cost
      Analysis" OR "Cost Utility" OR  "Cost Minimisation" OR "Cost
      Minimization" OR "Cost Effectiveness" OR "Cost Benefit" OR "Cost
      consequence" OR "Pharmacoeconomic" OR "Economic evaluation" OR
      "Economic analysis"))
AND   Keywords: "Cardiovascular disorders" OR "coronary disease"
AND   Year delimiter: 1995 to 2005
```

NHS EED

```
      All fields: prevention OR health promotion OR screening OR smoking OR
      Dietary intake OR exercise OR nutrition OR diet OR Physical activity
      OR Obesity OR weight management
AND   Subject Headings: Cardiovascular-Diseases OR Coronary-Disease
AND   Record type: Economic evaluations
AND   Date of publication: 1995 thru 2005
```

OHE HEED (Expert Search)

```
      AX [All data]: 'Coronary disease' OR 'coronary heart disease' OR
      'tobacco' OR 'Cardiovascular disease' OR 'Smoking' OR 'dietary intake'
      OR 'weight management' OR 'Dyslipidemia' OR 'physical activity' OR
      'hypercholesterolaemia' OR 'hypercholesterolemia' OR 'exercise' OR
      'obesity' OR 'diet' OR 'nutrition'
AND   TA [Technology assessed]: 'Screening' OR 'Prevention' OR 'Promotion'
      OR 'Pharmaceutical'
AND   JD[Journal date]:1995 thru 2005
```
