# Supplementary material for: The economics of primary prevention of cardiovascular disease – a systematic review of economic evaluations
Source: Cost Eff Resour Alloc. 2007 May 14;5:5. doi: 10.1186/1478-7547-5-5 (PMC1876202; doi:10.1186/1478-7547-5-5)
Supplement: Additional File 2 — Appendix 2 - Details of studies included in phase 2 assessment [file 1478-7547-5-5-S2.pdf]

Additional files

Appendix 2 – Details of studies included in phase 2 assessment

Health economic evaluations of the prevention of cardiovascular diseases – Intervention target: **OBESITY**; studies 1-4

**Author**	**Warren E**	**Hertzman P**	**Herman WH**	**Malone DC**
**Year of publication**	**2004**	**2005**	**2005**	**2005**
**Journal (Journal category)**	**Medical Decision Making** *(Health economics)*	**Pharmacoeconomics** *(Health economics)*	**Annals of internal medicine** *(Medicine)*	**Journal of managed care pharmacy** *(Health economics)*
**Title**	**Cost-effectiveness of Sibutramine in the treatment of obesity**	**The cost-effectiveness of Orlistat in a 1-year weight management programme for treating overweight and obese patients in Sweden**	**The cost-effectiveness of lifestyle modification or metformin in preventing type 2 diabetes in adults with impaired glucose tolerance**	**Cost-effectiveness of Sibutramine in the LOSE Weight Study: Evaluating the role of pharmacologic weight-loss therapy within a weight management program**
**Objective**	To report the incremental cost-utility of Sibutramine compared to diet and lifestyle advice for the treatment of obesity	To calculate the cost-effectiveness of Orlistat plus diet versus diet only for an obese an overweight population	To calculate the cost-effectiveness of lifestyle modification versus Metformin versus placebo	To compare the cost-effectiveness of Sibutramine plus a structured weight management program (WMP) versus WMP alone
**Intervention**	1-year treatment with 10mg (15mg in non-responders) Sibutramine	1-year treatment with three capsules of Orlistat per day in addition to diet	16-lesson core curriculum covering diet, exercise, and behavior modification taught by case managers on a one-on-one basis, followed by individual- and group sessions with manager on a monthly basis	Treatment with Sibutramine (not described in detail) plus WMP
**Comparator**	1-year treatment with diet and lifestyle advice	1.: 1-year diet only weight management<br>2.: No intervention	1.: Metformin 850mg/day for the 1st month, followed by 2x850mg/day for subsequent months<br>2.: Placebo	WMP: physician supervised, incl. 5 monitored care visits with a prevention specialist and attendance at 2 or more weight management seminars (10 classes each)
**Study setting**	United Kingdom (Europe)	Sweden (Europe)	USA (North America)	USA (North America)
**Study design**	Modeling	Modeling	Modeling	Randomized clinical trial
**Type of evaluation**	Cost-utility analysis	Cost-utility analysis	Cost-utility analysis	CEA* (clinical outcome)
**Intervention target**	Obesity	Obesity	Obesity	Obesity

Health economic evaluations of the prevention of cardiovascular diseases – Intervention target: **OBESITY**; studies 1-4

**Author**	**Warren E**	**Hertzman P**	**Herman WH**	**Malone DC**
**Type of intervention**	Clinical prevention:<br>*Pharmacotherapy*	Clinical prevention:<br>*Pharmacotherapy*	Clinical prevention:<br>*Health education*	Clinical prevention<br>*Pharmacotherapy*
**Target group (Gender, risk factor/co-morbidity, age)**	Male & female<br>Obese<br>Age: 30-74	Male & female<br>Obese<br>Additional CVD† risk factor<br>Age: ≥18	Male & female<br>Impaired glucose tolerance<br>Age: ≥25	Male & female<br>Obese<br>Age: ≥18
**Analytic horizon**	5 years	10 years	Lifetime	1 year
**Discounting & Base year**	Costs: 6%<br>Benefits: 1.5%<br>Base year: 2000	Costs: 3%<br>Benefits: 3%<br>Base year: Not adjusted to (2003)	Costs: 3%<br>Benefits: 3%<br>Base year: 2000	Not discounted<br>Base year: 2004
**Perspective**	Third party payer	Health care sector	Societal	Third party payer
**Benefit measurements**	Weight loss<br>Avoided CVD related incidents<br>Quality of life<br>QALYs‡	Weight loss<br>Avoided Diabetes<br>QALYs‡	Weight loss<br>QALYs‡	Weight loss
**Benefit results**	58.95 QALYs‡ gained by intervention compared to comparator	>10% reduction of weight:<br>Int.:44.8%; Diet: 24.5%<br>QALYs‡/ patient:<br>Int.: 0.1220; Diet: 0.0915	QALYs‡:<br>Lifestyle vs. Placebo: 0.57<br>Metformin vs. Placebo: 0.13	Interv.: -13.7 pounds (-6%)<br>Comp.: -5 pounds (-2.2%)
**Costs included**	Direct costs:<br>*Monthly drug costs, GP consultation, nurse consultation, drug induced side-effects*<br>Resource consumption stated: *No*<br>Indirect costs: *No*<br>Future costs (saved):<br>*Avoided Diabetes and CVD*†<br>Future costs (caused): *No*<br>Implementation costs: *No*	Direct costs:<br>*Medication, outpatient care, hospitalization*<br>Resource consumption stated: *No*<br>Indirect costs: *No*<br>Future costs (saved): T*reatment costs for diabetes*<br>Future costs (caused): *No*<br>Implementation costs: *No*	Direct costs:<br>*Screening, medical costs (also outside trial), side-effects, maintation costs*<br>Resource consumption stated: *No*<br>Indirect costs: *No*<br>Future costs (saved):<br>*Avoided Diabetes and CVD*†<br>Future costs (caused): *No*<br>Implementation costs: *Yes*	Direct costs:<br>*(only obesity related)*<br>*Outpatient visits, hospitalizations, professional service claims, prescriptions*<br>Resource consumption stated: *Yes*<br>Indirect costs: *No*<br>Future costs (saved): *No*<br>Future costs (caused): *No*<br>Implementation costs: *No*
**Cost results**	Interv.: £ 533,088<br>Comp.: £ 251,297	Interv.: € 720/patient<br>Diet: € 320/patient	Interv.: $ 4,967<br>Metformin: $ 3,748	Interv.: $ 408<br>Comp.: $ 31
**Outcome ratio**	ICER§: £ 4,780/ QALY‡	ICER§: €13,125/ QALY‡	Lifest. vs. Plac.: $ 8,790/QALY‡	Interv.: $ 32/ pound lost

Health economic evaluations of the prevention of cardiovascular diseases – Intervention target: **OBESITY**; studies 1-4

**Author**	**Warren E**	**Hertzman P**	**Herman WH**	**Malone DC**
			Metfo. vs. Plac.: $ 29,900/QALY‡	Comp.: $ 12/pound lost<br>ICER§ (Int.): $12/add. pound lost
**Methods to handle uncertainty**	Multi-way analysis<br>*Highly sensitive for the QALY‡ estimation of weight loss*	Probabilistic analysis<br>*90% of ICERs§ below €20,000/QALY‡*	Multi-way analysis	Multi-way analysis<br>*When all costs included, then ICER§ $194/add. pound lost*
**Sponsorship**	Industry	Not stated	Government	Industry

* Cost-effectiveness analysis; § Incremental cost-effectiveness ratio; ‡ Quality adjusted life year; † Cardio-vascular disease; ¶ Life years gained

Health economic evaluations of the prevention of cardiovascular diseases – Intervention target: **OBESITY**; studies 5-8

**Author**	**Lamotte M**	**Maetzel A**	**Wang LY**	**Foxcroft DR**
**Year of publication**	**2002**	**2003**	**2003**	**2005**
**Journal (Journal category)**	**Diabetes Care** *(Medicine)*	**Pharmacoeconomics** *(Health economics)*	**Obesity Research** *(Medicine)*	**Obesity Reviews** *(Medicine)*
**Title**	**A health economic model to assess the long-term effects and cost-effectiveness of Orlistat in obese type 2 diabetic patients**	**Economic evaluation of Orlistat in overweight and obese patients with type 2 diabetes mellitus**	**Economic analysis of a school-based obesity prevention program**	**Orlistat for the treatment of obesity: cost utility model**
**Objective**	To assess the long-term cost effectiveness of Orlistat	To estimate the economic value of pharmacological treatment of type 2 diabetes using Orlistat in addition to standard diabetes therapy and weight management	To assess the cost-effectiveness of “planet health”, a school based intervention designed to reduce obesity in youth of middle-school aged children	To assess the cost-utility of Orlistat treatment based on the prescription criteria from the NICE (for UK) and EMEA (Europ. Union) for obese patients
**Intervention**	2-year treatment with Orlistat (not defined in detail)	1-year treatment with Orlistat 3x120mg /day, weight management program and standard diabetic care	School based interdisciplinary curriculum among four major subject areas (language arts, math, science and social studies) and physical education. Focus: Decrease of TV viewing and consumption of high-fat food, increase of vegetable and fruit intake as well as physical activity	Treatment with Orlistat:<br>1.: Modeled for NICE criteria<br>2.: Modeled for EMEA criteria
**Comparator**	Placebo	Placebo, weight management program and stand. diabetic care	No intervention	Placebo
**Study setting**	Belgium (Europe)	USA (North America)	USA (North America)	United Kingdom (Europe)
**Study design**	Modeling	Modeling	Modeling	Modeling
**Type of evaluation**	CEA* (life years gained)	CEA* (clinical outcome)	Cost-utility analysis	Cost-utility analysis
**Intervention target**	Obesity	Obesity	Obesity	Obesity
**Type of intervention**	Clinical prevention: *Pharmacotherapy*	Clinical prevention: *Pharmacotherapy*	Health promotion: *Education*	Clinical prevention *Pharmacotherapy*
**Target group (Gender, risk factor/co-morbidity, age)**	Male & female<br>Obese<br>Type 2 diabetes<br>Age: Not stated	Male<br>Obese<br>Type 2 diabetes<br>Age: 52	Female<br>Age: 14-16	Male & female<br>Obese<br>Age: Not stated

Health economic evaluations of the prevention of cardiovascular diseases – Intervention target: **OBESITY**; studies 5-8

**Author**	**Lamotte M**	**Maetzel A**	**Wang LY**	**Foxcroft DR**
**Analytic horizon**	10 years	11 years	25 years (starting at age 40)	1 year
**Discounting & Base year**	Costs: 3%<br>Benefits: 0%<br>Base year: 2000	Costs: 3%<br>Benefits: 3%<br>Base year: 2001	Costs: 3%<br>Benefits: 3%<br>Base year: 1996	Not discounted<br>Base year: Not stated
**Perspective**	Patient	Not stated	Societal	Not stated
**Benefit measurements**	Life years gained	Avoided CVD related incidents<br>Event-free life years gained	Cases of adulthood obesity prevented<br>QALYs‡	QALYs‡
**Benefit results**	Interv.: 0.08 life years gained	Interv.: 0.13 event-free LYG¶	Cases prevented: 5.805<br>QALYs‡ gained: 4.13	(per 100 people)<br>NICE: 0.931 QALYs‡ gained<br>EMEA: 1.464
**Costs included**	Direct costs:<br>*Medical costs (Orlistat and diabetes treatment)*<br>Resource consumption stated: *No*<br>Indirect costs: *No*<br>Future costs (saved):<br>*Avoided CVD*†<br>Future costs (caused): *No*<br>Implementation costs: *No*	Direct costs:<br>*Medical costs, side-effects*<br>Resource consumption stated: *No*<br>Indirect costs: *No*<br>Future costs (saved):<br>*Avoided CVD*†<br>Future costs (caused): *No*<br>Implementation costs: *No*	Direct costs:<br>*Curriculum book, teacher training (trainer, assistant trainer, teacher's stipend, snacks), incentives for teacher*<br>Resource consumption stated: *Yes*<br>Indirect costs: *Productivity loss averted (human capital approach)*<br>Future costs (saved):<br>*Medical costs due to obesity*<br>Future costs (caused): *No*<br>Implementation costs: *No*	Direct costs:<br>*Prescription costs, consultation costs, pharmacological costs*<br>Resource consumption stated: *No*<br>Indirect costs: *No*<br>Future costs (saved): *No*<br>Future costs (caused): *No*<br>Implementation costs: *No*
**Cost results**	Interv.: € 17,180<br>Comp.: € 15,573	Interv.: $ 19,987<br>Comp.: $ 18,865	Direct costs: $ 33,677<br>Future costs (saved): $ 15,887<br>Produc. loss averted: $ 25,104	(per 100 people)<br>NICE: £ 22,744<br>EMEA: £ 27,823
**Outcome ratio**	ICER§: € 19,986/ life year gained	ICER§: $ 8327/ event-free LYG¶	Excl. productivity loss averted: $ 4305/QALY‡<br>Incl.: Cost saving ($ 7313)	NICE: £ 24,431/QALY‡<br>EMEA: £ 19,005/QALY‡
**Methods to handle uncertainty**	One-way analysis<br>*Highly sensitive for the estimated effect on HbA1c*	Probabilistic analysis<br>*95% of ICERs§ below $68,000/ event free life year gained*	Multi-way analysis<br>*Not tested for variations in the effect of the program*	Multi-way analysis<br>*Highly sensitive for QALY‡ gain assumed and for prescription delivery assumed (physician vs. nurse)*
**Sponsorship**	Industry	Industry	Government	Industry

* Cost-effectiveness analysis; § Incremental cost-effectiveness ratio; ‡ Quality adjusted life year; † Cardio-vascular disease; ¶ Life years gained

Health economic evaluations of the prevention of cardiovascular diseases – Intervention target: **OBESITY**; studies 9-12

**Author**	**Siggaard R**	**Martin LF**
**Year of publication**	**1996**	**1995**
**Journal (Journal category)**	**Obesity Research** *(Medicine)*	**Surgery** *(Medicine)*
**Title**	**Weight loss during 12 weeks' ad libitum carbohydrate rich diet in overweight and normal weight subjects at a Danish work site**	**Comparison of the costs associated with medical and surgical treatment of obesity**
**Objective**	To estimate the costs and effects of a carbohydrate rich diet on total body weight, lean body mass and fat mass in a group of healthy subjects.	To assess the long term costs and outcomes for gastric bypass surgery in obese patients versus a diet
**Intervention**	Receiving a dietary guideline containing carbohydrate rich/ low fat recipes; once a week instructions (lectures and material) focusing on physiology, appetite regulation, macronutrient balance and how to read food labels	-Roux-en-Y gastric bypass, connecting a 40cm Roux limb of jejunum to a 20ml gastric pouch<br>-Very-low-calories-diet for week 1-8; quarterly newsletter; list of peers for contact; support group which met monthly
**Comparator**	No intervention, after 12 weeks receiving of guidelines on how to change diet	Diet advice in weekly meetings for 1 ½ years:4 weeks orientation; 12 weeks supplement fast; 10 weeks transitional phase; 52 weeks maintenance phase; education in: nutrition, exercise, modification of lifestyle, eating behavior
**Study setting**	Denmark (Europe)	USA (North America)
**Study design**	Prospective observational trial	Retrospective observational analysis
**Type of evaluation**	CEA* (clinical outcome)	CEA* (clinical outcome)
**Intervention target**	Obesity	Obesity
**Type of intervention**	Clinical prevention:<br>*Health education*	Clinical prevention:<br>*Surgery*
**Target group (Gender, risk factor/co-morbidity, age)**	Male & female<br><br>Age: Not stated (mean 41.5 years)	Male & female<br>Obese<br>Age: 18-71
**Analytic horizon**	1 year (52 weeks)	6 years

Health economic evaluations of the prevention of cardiovascular diseases – Intervention target: **OBESITY**; studies 9-12

**Author**	**Siggaard R**	**Martin LF**	
**Discounting & Base year**	Not discounted<br><br>Base year: Not stated	Not discounted<br><br>Base year: Not stated	
**Perspective**	Not stated	Not stated	
**Benefit measurements**	Weight loss	Weight loss	
**Benefit results**	Weight loss in subjects being:<br>Overweight / Normal weight<br>28% >5kg 0%<br>41% 2.5-5kg 25%<br>31% 0-2.5kg 50%<br>0% weight gain 25%	Subjects considered "Success" after 6 years:<br>Interv.: 88%<br>Comp.: 29%<br>(⅓ of weight reduction considered "success")	
**Costs included**	Direct costs:<br>*Materials, supervision, administration by one employee*<br>Resource consumption stated: *No*<br>Indirect costs: *No*<br>Future costs (saved): No<br>Future costs (caused): *No*<br>Implementation costs:<br>*Organization, instruction*	Direct costs:<br>*Physical examination, psychological evaluation, laboratory tests, EKG/X-ray, other medical charges, behavior training, hospital charges*<br>Resource consumption stated: *No*<br>Indirect costs: *No*<br>Future costs (saved): *No*<br>Future costs (caused): *No*<br>Implementation costs: *No*	
**Cost results**	Not reported separately	Interv.: $24,000<br>Comp.: $3,000	
**Outcome ratio**	$ 14.7 /kg lost per person<br>$ 11.9 /1% overweight reduction per person	Only by graph:<br>Interv.: ca. $760/ pound lost<br>Comp.: ca. $1,550/ pound lost	
**Methods to handle uncertainty**	Multi-way analysis	No handling of uncertainty stated	
**Sponsorship**	Industry	Not stated	

* Cost-effectiveness analysis; § Incremental cost-effectiveness ratio; ‡ Quality adjusted life year; † Cardio-vascular disease; ¶ Life years gained

Health economic evaluations of the prevention of cardiovascular diseases – Intervention target: **DIETARY INTAKE**; studies 1-4

**Author**	**Sikand G**	**Plans Rubio P**	**Tosteson ANA**	**Prosser LA**
**Year of publication**	**2000**	**1997**	**1997**	**2000**
**Journal (Journal category)**	**Journal of the American Dietetic Association** *(Medicine)*	**Public Health** *(Public health)*	**Circulation** *(Medicine)*	**Annals of internal medicine** *(Medicine)*
**Title**	**Dietitian intervention improves lipid values and saves medication costs in men with combined hyperlipidemia and a history of Niacin noncompliance**	**Cost-effectiveness of dietary treatment of hypercholesterolemia in Spain**	**Cost-effectiveness of populationwide educational approaches to reduce serum cholesterol levels**	**Cost-effectiveness of cholesterol-lowering therapies according to selected patient characteristics**
**Objective**	To measure, whether medical nutrition therapy (MNT) administered by registered dietitians could lead to beneficial medical and financial outcomes	To assess the cost-effectiveness of dietary treatment in Spain	To simulate the cost-effectiveness of a populationwide educational intervention to lower serum cholesterol in the US population	To evaluate how the cost-effectiveness ratios of cholesterol-lowering therapies vary according to different risk factors
**Intervention**	1-year treatment with MNT before using Statins for therapy	Diet supported by medical visits and laboratory testing. American Heart Association phase-I-diet: 30% of calories as fat, 55% as carbohydrate and 15% of protein. Less than 300mg cholesterol/day	Education through mass media campaigns, including television, radio and newspaper and other printed material. Direct education through community activities and face-to face instructions.	Step-I-diet: cholesterol low diet
**Comparator**	Treatment with Statins immediately	No intervention	No intervention	1.: Treatment with Statins<br>2.: No preventive treatment
**Study setting**	USA (North America)	Spain (Europe)	USA (North America)	USA (North America)
**Study design**	Prospective observational trial	Modeling	Modeling	Modeling
**Type of evaluation**	Cost-consequence analysis	CEA* (clinical outcome)	CEA* (life years gained)	Cost-utility analysis
**Intervention target**	Dietary intake	Dietary intake	Dietary intake	Dyslipidemia (Dietary intake)
**Type of intervention**	Clinical prevention: *Health education*	Clinical prevention: *Health education*	Health promotion: *Education*	Clinical prevention *Health education*
**Target group (Gender, risk factor/co-morbidity, age)**	Male<br>Combined hyperlipidemia<br>Niacin noncompliance<br>Age: 60 ± 10.1	Male & female<br>Hypercholesterolemia<br>Age: 35-64	Male & female<br>Age: 35-84	Male & female<br>Dyslipidemia<br>Age: 35-84

Health economic evaluations of the prevention of cardiovascular diseases – Intervention target: **DIETARY INTAKE**; studies 1-4

**Author**	**Sikand G**	**Plans Rubio P**	**Tosteson ANA**	**Prosser LA**
**Analytic horizon**	1 year	Lifetime	25 years	30 years
**Discounting & Base year**	Not discounted<br><br>Base year: Not stated	Costs: 5%<br>Benefits: 5%<br>Base year: 1990	Costs: 5%<br>Benefits: 5%<br>Base year: 1993	Costs: 3%<br>Benefits: 3% (diet), 6% (Statin therapy, because of side-effects)<br>Base year: 1997
**Perspective**	Not stated	Societal	Not stated	Societal
**Benefit measurements**	Blood lipid level	Blood lipid level<br>Avoided CVD[†] related incidents<br>Incident free life years gained	Life years gained	Blood lipid level<br>QALYs[‡]
**Benefit results**	Interv.: 15 of 30 patients treated to NCEP guidelines lipid level	Incident free life years gained:<br>Men: 12-141, Women: 6-56	≈ 624,000 life years gained	Not stated separately
**Costs included**	Direct costs:<br>*Counseling by dietitian, medical costs for statins*<br>Resource consumption stated: *No*<br>Indirect costs: *No*<br>Future costs (saved): *No*<br>Future costs (caused): *No*<br>Implementation costs: *No*	Direct costs:<br>*Medical visits, cholesterol analysis*<br>Resource consumption stated: *No*<br>Indirect costs: *No*<br>Future costs (saved):<br>*Avoided CVD*[†]<br>Future costs (caused): *No*<br>Implementation costs:<br>*More medical visits during 1st year*	Direct costs:<br>*Program costs*<br>Resource consumption stated: *No*<br>Indirect costs: *No*<br>Future costs (saved):<br>*Avoided CVD*[†]<br>Future costs (caused): *No*<br>Implementation costs:<br>*Higher program costs during 1st year*	Direct costs:<br>*Medication, physician visits*<br>Resource consumption stated: *No*<br>Indirect costs: *Time for physician visit (human capital approach)*<br>Future costs (saved):<br>*Avoided CVD*[†]<br>Future costs (caused): *No*<br>Implementation costs:<br>*More medical visits and laboratory testing during 1st year*
**Cost results**	Interv.: $ 13,545<br>Comp.: $ 40,994	1st year: $ 271/ person<br>After 1st year: $ 92-123/ person	≈ $ 2.1 billion	Not stated separately
**Outcome ratio**	Not stated	Varies by sex, age & lipid level:<br>$ 16,143/ incident free life year<br>(45-49 year, male, chol.300mg/dl)	$ 3,200/ life year gained	Diet vs. No interv. (ICER):<br>$ 1,900 - $ 500,000/ QALY<br>Statins vs. Diet (ICER):<br>$ 54,000 - $ 1,400,000/ QALY
**Methods to handle uncertainty**	No handling of uncertainty stated	One-way analysis<br>*Highly sensitive to assumption of cholesterol reduction*	Multi-way analysis<br>*Highly sensitive to assumption of cholesterol reduction*<br>Threshold analysis:<br>(costs per person per year)	Multi-way analysis<br>*Results not stated*<br>Threshold analysis:<br>(cost-effective in risk subgroups)<br>If $ 50,000/ QALY<br>(Diet: 159/240; Statins 0/240)

Health economic evaluations of the prevention of cardiovascular diseases – Intervention target: **DIETARY INTAKE**; studies 1-4

**Author**	**Sikand G**	**Plans Rubio P**	**Tosteson ANA**	**Prosser LA**
			if $50,000/LYG[¶]<br>(Costs: < $ 20)	If $ 100,000/ QALY<br>(Diet: 210/240; Statins 62/240)
**Sponsorship**	Foundation	Not stated	Government	Foundation

* Cost-effectiveness analysis; [§] Incremental cost-effectiveness ratio; [‡] Quality adjusted life year; [†] Cardio-vascular disease; [¶] Life years gained

Health economic evaluations of the prevention of cardiovascular diseases – Intervention target: **DIETARY INTAKE**; studies 5-8

**Author**	**Tice JA**	**Lindgren P**	**Olsen J**	**Delahanty LM**
**Year of publication**	**2001**	**2003**	**2005**	**2001**
**Journal (Journal category)**	**JAMA** *(Medicine)*	**Preventive Medicine** *(Public health)*	**Int Journal of Technology Assessment in Health Care** *(Health economics)*	**Journal of the American Dietary association** *(Medicine)*
**Title**	**Cost-effectiveness of vitamin therapy to lower plasma homocysteine levels for the prevention of coronary heart disease**	**Cost-effectiveness of primary prevention of coronary heart disease through risk factor intervention in 60-year-old men from the county of Stockholm- a stochastic model of exercise and dietary advice**	**Cost-effectiveness of nutritional counseling for obese patients at risk of ischemic heart disease**	**Clinical and cost outcomes of medical nutrition therapy for hypercholesterolemia**
**Objective**	The cost-effectiveness of a grain fortification with folic acid and additional vitamin supplementation	To estimate the cost-effectiveness of different risk factor interventions in 60-year-old healthy men	To compare the costs and effects of providing nutritional counseling by a general practitioner (GP) versus a dietitian	To compare the cost-effectiveness of a cholesterol lowering protocol implemented by dietitians versus advice by physicians
**Intervention**	Diet with 1 mg folic acid and 0.5 mg cyanocobalamin (Vitamin) per day (through enriched food by legislation and pharmacotherapy)	"Dietary advice": Advice by a physician and visit to a dietitian for individual counseling	"GP": Counseling in terms of general advice and delivery of commercially available written information on healthy diet<br>1stsession:30min Following:12min	Medical nutrition therapy (MNT):<br>2-3 meeting with dietitian, if blood lipid level meets not target range at end of sessions, then another 2-3 meetings
**Comparator**	1.: Diet with enriched grains that raise the intake of folic acid by 100 µg/day (through enriched food by legislation)<br>2.: No intervention	1.: "Exercise": Advice by a physician to maintain a prepared activity log and the opportunity to join an exercise group<br>2.: "Dietary advice" & "Exercise"<br>3.: No intervention	"Dietitian": Individual counseling and exercises focusing on good nutrition, food shopping, cooking, and meal planning. Advice on dietary restrictions (reducing total, fat and cholesterol intake)<br>1st session: 1h ; Following: 30min	Usual care (UC):<br>Customary cholesterol advice by the healthcare provider in an ambulatory setting
**Study setting**	USA (North America)	Sweden (Europe)	Denmark (Europe)	USA (North America)
**Study design**	Modeling	Modeling	Combination Trial-Modeling	Randomized clinical trial
**Type of evaluation**	Cost-utility analysis	CEA* (life years gained)	CEA* (life years gained)	CEA* (clinical outcome)
**Intervention target**	Dietary intake	Dietary intake	Dietary intake	Dietary intake
**Type of intervention**	Health promotion:<br>*Legislation (Pharmacotherapy)*	Clinical prevention:<br>*Health Education*	Clinical prevention:<br>*Health Education*	Clinical prevention<br>*Health education*

Health economic evaluations of the prevention of cardiovascular diseases – Intervention target: **DIETARY INTAKE**; studies 5-8

**Author**	**Tice JA**	**Lindgren P**	**Olsen J**	**Delahanty LM**
**Target group (Gender, risk factor/co-morbidity, age)**	Male & female<br>Age: 35-85	Male<br>Age: 60	Male & female<br>Obesity, dyslipidemia or diabetes<br>Age: Not stated	Male & female<br>Hypercholesterolemia<br>Age: 21-65
**Analytic horizon**	10 years	Lifetime	Lifetime	6 month
**Discounting & Base year**	Costs: 3%<br>Benefits: 3%<br>Base year: 1997	Costs: 3%<br>Benefits: 3%<br>Base year: 2000	Costs: No discounting stated<br>Benefits: 5%<br>Base year: 2001	Not discounted<br>Base year: Not stated
**Perspective**	Health care sector	Societal	Not stated	Not stated
**Benefit measurements**	Avoided CVD[†] related incidents<br>QALYs[‡]	Blood lipid level<br>Blood pressure<br>Life years gained	Life years gained	Blood lipid level<br>Change of behavior<br>Weight loss
**Benefit results**	Change in % of CVD[†] incidents:<br>1. Comp. vs. No interv.:<br>- 13% (men), -8% (women)<br>Others not stated	No interv.: 13.229 years survival<br>Diet: 13.252 years survival<br>Exercise: 13.246 years survival<br>D & E : 13.245 years survival	Interv. vs. No interv.:<br>0.0919 life years gained<br>Comp. vs. No interv.:<br>0.0274 life years gained	Total & LDL cholesterol level:<br>Interv.: - 6%<br>Comp.: - 2%
**Costs included**	Direct costs:<br>*Vitamin costs, folic acid, laboratory costs*<br>Resource consumption stated: *No*<br>Indirect costs: *No*<br>Future costs (saved):<br>*Myocardial infarction and death*<br>Future costs (caused): *No*<br>Implementation costs: *No*	Direct costs:<br>*Health care expenditure as a result of disease or intervention*<br>Resource consumption stated: *No*<br>Indirect costs: *Loss of production averted (human capital approach)*<br>Future costs (saved): *No*<br>Future costs (caused):<br>*Future consumption*<br>Implementation costs: *No*	Direct costs:<br>*Time spent by GP or dietitian*<br>Resource consumption stated: *No*<br>Indirect costs: *Patients time spent at visit (human capital approach)*<br>Future costs (saved): *No*<br>Future costs (caused): *No*<br>Implementation costs: *No*	Direct costs:<br>*Counseling*<br>Resource consumption stated: *No*<br>Indirect costs: *No*<br>Future costs (saved): *No*<br>Future costs (caused): *No*<br>Implementation costs: *No*
**Cost results**	Not stated for primary prevention	No interv.: SEK 1,280,678<br>Diet: SEK 1,283,570<br>Exercise: SEK 1,283,746<br>D & E : SEK 1,283,900	Interv.: DKK 755<br>Comp.: DKK 1,642	Interv. vs. Comp.: $ 217
**Outcome ratio**	Interv. vs. 1. Comp. (ICER):<br>Cost saving (men) –<br>$ 830,000/QALY[‡] (women)	Diet vs. No interv.(ICER[§]):<br>SEK 127,065/ life year gained	Interv.: DKK 11,978/ LYG[¶]<br>Comp.: DKK 73,339/ LYG[¶]	Intervention:<br>$ 36/ 1% reduction of cholesterol level

Health economic evaluations of the prevention of cardiovascular diseases – Intervention target: **DIETARY INTAKE**; studies 5-8

**Author**	**Tice JA**	**Lindgren P**	**Olsen J**	**Delahanty LM**
**Methods to handle uncertainty**	Multi-way analysis<br>*Only few results stated*	No handling of uncertainty stated	Probabilistic analysis<br>*Dietitian counseling highly sensitive*	No handling of uncertainty stated
**Sponsorship**	Not stated	Foundation	Not stated	Foundation

* Cost-effectiveness analysis; [§] Incremental cost-effectiveness ratio; [‡] Quality adjusted life year; [†] Cardio-vascular disease; [¶] Life years gained

Health economic evaluations of the prevention of cardiovascular diseases – Intervention target: **DIETARY INTAKE**; studies 9-12

**Author**	**Selmer RM**	**Brannon SD**	**Hornberger J**	**Tomson Y**
**Year of publication**	**2000**	**2003**	**1998**	**1995**
**Journal (Journal category)**	**Journal of Epidemiology and Community Health** *(Public health)*	**American Journal of Public Health** *(Public health)*	**American Journal of Public Health** *(Public Health)*	**Journal of Internal Medicine** *(Medicine)*
**Title**	**Cost and health consequences of reducing the population intake of salt**	**The cost-effectiveness of alternative methods of nutrition education for hypercholesterolemic children**	**A cost-benefit analysis of a cardiovascular disease prevention trial, using folate supplementation as an example**	**The cost and effects of two different lipid intervention programmes in primary health care**
**Objective**	To estimate health and economic consequences of different strategies to reduce the daily intake of salt	Compare the cost-effectiveness, from the family's perspective , of a parent-child auto tutorial (PCAT) compared to counseling	To estimate the cost-benefit and sample size of a placebo controlled trial of folate supplementation to prevent CVD[†]	To compare the cost and effects of two different interventions for non-pharmacological treatment of hypercholesterolemia
**Intervention**	Information campaigns, developing industry food with less salt, legislation for the declaration of salt content of food, taxation/subsidizing of food with high/little salt content	PCAT: Audio taped stories and accompanying picture books including follow up paper-and-pencil activities for children as well as a manual for parents	To treat patients with elevated homocysteine levels with folate-acid	Two visits to a physician and three to a dietitian. At dietitian: 1st visit: Individual analysis of eating pattern and forming of solutions; 2nd visit: Including spouse into counseling ; 3rd visit: Exercise in small groups into grocery stores to identify low fat food
**Comparator**	No intervention	Counseling: Scheduled 45-60 min session with a registered dietitian	Treatment with placebo	Sending a letter to General practitioner containing booklet about simple diet information
**Study setting**	Norway (Europe)	USA (North America)	USA (North America)	Sweden (Europe)
**Study design**	Modeling	Randomized clinical trial	Modeling	Randomized clinical trial
**Type of evaluation**	CEA* (life years gained)	CEA* (clinical outcome)	Cost-benefit analysis	Cost-consequence analysis
**Intervention target**	Dietary intake	Dietary intake (Dyslipidemia)	Dietary intake	Dietary intake (Dyslipidemia)
**Type of intervention**	Health promotion: *Legislation*	Clinical prevention: *Health Education*	Clinical prevention: *Pharmacotherapy*	Clinical prevention *Health education*
**Target group (Gender, risk factor/co-morbidity, age)**	Male & female<br><br>Age: ≥40	Male & female<br>Hyperlipidemia<br>Age: 4-10	Male & female<br>Elevated homocysteine level<br>Age: 35-84	Male & female<br>Hypercholesterolemia<br>Age: 25-54

Health economic evaluations of the prevention of cardiovascular diseases – Intervention target: **DIETARY INTAKE**; studies 9-12

**Author**	**Selmer RM**	**Brannon SD**	**Hornberger J**	**Tomson Y**
**Analytic horizon**	Lifetime	1 year (12 months)	15 years	1 year
**Discounting & Base year**	Costs: 5%<br>Benefits: 5%<br>Base year: 1997	No discounting stated<br>Base year: 1992	Costs: 3%<br>Benefits: 3%<br>Base year: 1996	Not discounted<br>Base year: 1993
**Perspective**	Societal	Family	Not stated	Not stated
**Benefit measurements**	Life years gained	Blood lipid level	Avoided CVD related incidents<br>Life years gained<br>QALYs[‡]<br>Willingness to pay	Blood lipid level
**Benefit results**	Men: 1.8 months gained<br>Women: 1.4 months gained	% reduction of calories in fat:<br>PCAT: 1.6; Counseling: 2.6<br>mg/dl reduction of LDL:<br>PCAT: 5.84; Counseling: 7.04	1 QALY assumed to be $ 50,000 by willingness to pay<br>No further results stated	Serum cholesterol level difference in mmol/L:<br>Interv.: - 0.24<br>Comp.: - 0.27
**Costs included**	Direct costs: *Campaign*<br>Resource consumption stated: *No*<br>Indirect costs: *productivity gain, welfare loss by taxes (friction costs approach)*<br>Future costs (saved):<br>*Avoided CVD*<br>Future costs (caused): *Hospital costs in future life years*<br>Implementation costs: *Development costs for new products during the 1st year*	Direct costs:<br>*Screening, laboratory testing, intervention costs*<br>Resource consumption stated: *No*<br>Indirect costs: parents loss of time (human capital approach)<br>Future costs (saved): *No*<br>Future costs (caused): *No*<br>Implementation costs: *No*	Direct costs:<br>*Folate-acid per year,laboratory testing, medical care*<br>Resource consumption stated: *No*<br>Indirect costs: *No*<br>Future costs (saved):<br>*Avoided CVD*<br>Future costs (caused): *Cost per asymptomatic patient/year*<br>Implementation costs:<br>*Research cost in the first years*	Direct costs:<br>*Physician visits, dietitian visits, laboratory nurse, laboratory tests, traveling expenses*<br>Resource consumption stated: *No*<br>Indirect costs: T*ime lost by patients due to visits (human capital approach)*<br>Future costs (saved): *No*<br>Future costs (caused): *No*<br>Implementation costs: *No*
**Cost results**	Costs: $ 625,000,000<br>Avoided costs: $ 862,000,000<br>Total - $ 117,000,000	PCAT: $ 208.08<br>Couns.: $ 213.28	Not stated separately	Cost per subject:<br>Interv.: SEK 3,614<br>Comp.: SEK 753
**Outcome ratio**	Cost-saving	Per % of calories in fat reduction:<br>PCAT: $ 130.05; Couns.: $ 82.03<br>Per mg/dl LDL-reduction<br>PCAT: $ 35.63 Couns.: $ 30.30	After 15 years cost benefit saving will be ≈ $ 11 billion	Not stated
**Methods to handle uncertainty**	One way analysis<br>*Highly sensitive for the assumed blood*	No handling of uncertainty stated	Multi-way analysis<br>*Highly sensitive to assumed*	No handling of uncertainty stated

Health economic evaluations of the prevention of cardiovascular diseases – Intervention target: **DIETARY INTAKE**; studies 9-12

**Author**	**Selmer RM**	**Brannon SD**	**Hornberger J**	**Tomson Y**
	pressure reduction		*willingness-to-pay and to assumed effect on survival*	
**Sponsorship**	Government	Government	Not stated	Not stated

* Cost-effectiveness analysis; § Incremental cost-effectiveness ratio; ‡ Quality adjusted life year; † Cardio-vascular disease; ¶ Life years gained

Health economic evaluations of the prevention of cardiovascular diseases – Intervention target: **DIETARY INTAKE**; studies 13-14

**Author**	**Tsai AG**	**Cox RH**
**Year of publication**	**2005**	**2003**
**Journal**<br>**(Journal category)**	**Obesity Research**<br>*(Medicine)*	**Journal of the American Dietetic Association**<br>*(Medicine)*
**Title**	**Cost-effectiveness of a low-carbohydrate diet and a standard diet in severe obesity**	**A video lesson series is effective in changing the dietary intakes and food related behaviors of low-income homemakers**
**Objective**	To compare the cost-effectiveness of standard diet versus "low-carbohydrate" diet in severely obese patients	To examine the relative cost-effectiveness of a self-administered video series in delivering nutrition education to low-income home makers
**Intervention**	"Low-carbohydrate diet":<br>1st month: weekly counseling<br>Month 2-6: monthly counseling<br>Advice to consume <30g/day carbohydrate (1/10 of recommendation for 70kg male)	"Video group": 12 videotaped lessons plus handout; telephone discussions to encourage persons; three visits by para-professionals to conduct hands-on activities; and 2 visits for data collection
**Comparator**	NCEP Step-I-Diet:<br>Advice to restrict calories, total fat and total cholesterol intake.<br>Same counseling scheme as above	"Traditional group": 12 lessons delivered face-to-face or in small groups (once a week) by para-professionals; hands-on activities during sessions<br>Both groups use the "Eating right is Basic, 3rd Edition"-principles, containing advice on the "food pyramid", food preparation and safety, meal planning and budgeting, healthful breakfast and snacks
**Study setting**	USA (North America)	USA (North America)
**Study design**	Randomized clinical trial	Randomized clinical trial
**Type of evaluation**	Cost-utility analysis	Cost-minimization analysis
**Intervention target**	Dietary intake (Obesity)	Dietary intake
**Type of intervention**	Clinical prevention:<br>*Health education*	Clinical prevention:<br>*Health education*
**Target group (Gender, risk factor/co-morbidity, age)**	Male & female<br>Obese<br>Age: 54±9	Female<br><br>Age: 15-52
**Analytic horizon**	1 year	Not clearly stated<br>("end of lesson" approx. 3-4 months)

Health economic evaluations of the prevention of cardiovascular diseases – Intervention target: **DIETARY INTAKE**; studies 13-14

**Author**	**Tsai AG**	**Cox RH**	
**Discounting & Base year**	Not discounted<br><br>Base year: Not stated	Not discounted<br><br>Base year: Not stated	
**Perspective**	Societal	Not stated	
**Benefit measurements**	Quality of life (SF-36)<br>QALYs‡	Change of behavior	
**Benefit results**	QALYs‡ ("experienced")<br>Interv.: 0.64<br>Comp.: 0.61<br>*No base value stated*<br>*Difference not statistically significant (p=.17)*	PSBC (Pennsylvania State behavioral checklist) Factor Score change:<br>Interv.: +0.53 Comp.: +0.42<br>*Interv. vs. Comp: (p= .2357) not significant*<br>➔ *assumed equal benefits*	
**Costs included**	Direct costs: *Counseling, other therapies used by patient (Diet costs excluded)*<br>Resource consumption stated: *No*<br>Indirect costs: *Lost work time (human capital approach)*<br>Future costs (saved): *No*<br>Future costs (caused): *No*<br>Implementation costs: *No*	Direct costs:<br>*Para professional time (phone calls, visits, travel), travel costs, lesson materials (flipchart with handouts, video masters, video copies, drop-off envelopes, phone)*<br>Resource consumption stated: *Yes*<br>Indirect costs: *No*<br>Future costs (saved): *No*<br>Future costs (caused): *No*<br>Implementation costs: *No*	
**Cost results**	Direct costs:<br>Interv.: $ 6,742 Comp.: $ 6,249<br>Indirect costs:<br>Interv.: $ 224 Comp.: $ 176	Interv.: $ 4,820<br>Comp.: $ 13,463<br>*(Implementation costs for the video making explicitly excluded)*	
**Outcome ratio**	Point estimate (ICER) Interv.:<br>$ -1,225/QALY‡	Intervention costs only 36% of the comparator with no change in benefit	
**Methods to handle uncertainty**	Probabilistic analysis<br>*Confidence interval undefined*<br>*72.4% of ICERs below $ 50,000/QALY‡*	Multi-way analysis	
**Sponsorship**	Government	Not stated	

* Cost-effectiveness analysis; § Incremental cost-effectiveness ratio; ‡ Quality adjusted life year; † Cardio-vascular disease; ¶ Life years gained

Health economic evaluations of the prevention of cardiovascular diseases – Intervention target: **PHYSICAL INACTIVITY**; studies 1-4

**Author**	**Lowensteyn I**	**Sevick MA**	**Stevens W**	**Munro JF**
**Year of publication**	**2000**	**2000**	**1998**	**2004**
**Journal (Journal category)**	**Journal of Cardiopulmonary Rehabilitation** *(Medicine)*	**American Journal of Preventive Medicine** *(Public health)*	**British Journal of Sports Medicine** *(Medicine)*	**Journal of Epidemiology and Community Health** *(Public Health)*
**Title**	**The cost-effectiveness of exercise training for the primary and secondary prevention of cardiovascular disease**	**Cost-effectiveness of lifestyle and structured exercise interventions in sedentary adults: results of project ACTIVE**	**Cost-effectiveness of a primary care based physical activity intervention in 45-74 year old men and women: a randomised controlled trial**	**Cost-effectiveness of a community based exercise programme in over 65 year olds: cluster randomised trial**
**Objective**	To estimate the long term cost-effectiveness of exercise training on improving cardio-vascular risk factors	To evaluate the cost-effectiveness of two interventions approaching change in physical activity and cardiorespiratory fitness	To assess the cost-effectiveness of a primary care based intervention aimed at increasing the physical activity in inactive people	To assess the cost-effectiveness of a community based exercise program as a population wide public health intervention
**Intervention**	Supervised group exercise classes at least 3x per week for 30min per session within 65% to 85% of an individuums heart rate	Lifestyle intervention: Teaching of behavioral skills to increase physical activity by integrating moderate-intensity physical activity into daily lives. First 6 month: weekly and later bi-weekly meeting with facilitators	Invitation to a consultation with an exercise development officer and offer of a personalized 10 week program to increase activity combining home-based and leisure center activities	Offer to attend locally organized, free exercise classes (2x/ week, 75min each). Content: Improving joint mobility, muscle strength and endurance, balance coordination and cardio- fitness combined with "fun" activities
**Comparator**	1.: Unsupervised walking program performed outdoors or in the local shopping mall at the same intensity<br>2.: No intervention	Structured intervention: Receiving a typical exercise prescription and exercising at a state-of-the-art facility under supervision of a trainer. Participants received a motivational readiness manual	Sending information to participants with information about local leisure centers	No intervention
**Study setting**	Canada (North America)	USA (North America)	United Kingdom (Europe)	United Kingdom (Europe)
**Study design**	Modeling	Randomized clinical trial	Randomized clinical trial	Randomized clinical trial
**Type of evaluation**	CEA* (life years gained)	CEA* (clinical outcome)	CEA* (clinical outcome)	Cost-utility analysis
**Intervention target**	Physical inactivity	Physical inactivity	Physical inactivity	Physical inactivity
**Type of intervention**	Clinical prevention: *Health Education*	Clinical prevention: *Health Education*	Clinical prevention: *Health Education*	Clinical prevention *Health education*

Health economic evaluations of the prevention of cardiovascular diseases – Intervention target: **PHYSICAL INACTIVITY**; studies 1-4

**Author**	**Lowensteyn I**	**Sevick MA**	**Stevens W**	**Munro JF**
**Target group (Gender, risk factor/co-morbidity, age)**	Male & female<br><br>Age: 35-74	Male & female<br>Obese, sedentary<br>Age: 35-60	Male & female<br>Sedentary<br>Age: 45-74	Male & female<br>Sedentary<br>Age: >65
**Analytic horizon**	Lifetime	2 years	8 months	2 years
**Discounting & Base year**	Costs: 3%<br>Benefits: 3%<br>Base year: 1996	Costs: 5%<br>Benefits: No discounting stated<br>Base year: Not stated	Not discounted<br><br>Base year: Not stated	Not discounted<br><br>Base year: 2003/2004
**Perspective**	Not stated	Not stated	Not stated	Third party payer
**Benefit measurements**	Blood lipid level<br>Blood pressure<br>Life years gained	Weight loss<br>Blood pressure<br>Change of behavior	Change of behavior	Quality of life (SF-36)<br>QALYs‡
**Benefit results**	Interv. vs. No interv.: 0.70 LYG¶<br>1.Com. vs. No interv.: 0.70 LYG¶<br>*(Adherence of 100% assumed)*	Various, for example: Systolic blood pressure reduction (mmHg)<br>Interv.: -3.63; Comp.: -3.26	Reduction of sedentary (< 4x20min activity/week) people:<br>Interv. vs. Comp.: -10.6%	Interv. vs. No interv.:<br>0.011 QALYs‡ gained per person
**Costs included**	Direct costs: *Program, proper clothing and shoes*<br>Resource consumption stated: *No*<br>Indirect costs: *No*<br>Future costs (saved): *Avoided CVD*<br>Future costs (caused): *No*<br>Implementation costs: *Higher costs during the 1st year*	Direct costs: *Staff, computer tracking system, curriculum materials, facilities, health club memberships, postage*<br>Resource consumption stated: *No*<br>Indirect costs: *No*<br>Future costs (saved): *No*<br>Future costs (caused): *No*<br>Implementation costs: *Development costs for 1st cohort*	Direct costs:<br>*Postage, stationery, administration, exercise development officer*<br>Resource consumption stated: *No*<br>Indirect costs: *No*<br>Future costs (saved): *No*<br>Future costs (caused): *No*<br>Implementation costs: *Equipment*	Direct costs: *Running an exercise, recruitment, administration, hire of halls, payments to exercise leaders, refreshments*<br>Resource consumption stated: *Yes*<br>Indirect costs: *No*<br>Future costs (saved): *No*<br>Future costs (caused): *No*<br>Implementation costs: *Startup costs annualized over 5 years*
**Cost results**	1.Comp.: $ 13,234<br>*(Adherence of 100% assumed)*<br>No interv.: $ 13,416<br>Interv.: Not stated	Inter.:$ 17.5 per participant/month<br>Com.:$ 49.3 per participant/month	Inter.: £ 24,044	Total: € 128,302<br>Per session: € 125.78<br>Per participant: € 9.06
**Outcome ratio**	Interv.: $ 21,131 - $ 87,166/ LYG¶<br>1.Comp.: $ 345 - $ 11,191/ LYG¶<br>(Adherence assumed: 50% during 1st year;30% for subsequent years)	Systolic blood pressure reduction (addl. mmHg reduction)<br>Interv.: $ 3/per participant./month;<br>Comp.: $ 10/per participant/month	Costs per person moved out of sedentary lifestyle (ICER§):<br>Interv.: £ 623	Interv. vs. No interv. (ICER§)<br>€ 17,174/ QALY

Health economic evaluations of the prevention of cardiovascular diseases – Intervention target: **PHYSICAL INACTIVITY**; studies 1-4

**Author**	**Lowensteyn I**	**Sevick MA**	**Stevens W**	**Munro JF**
**Methods to handle uncertainty**	One way analysis<br>*Highly sensitive for assumed adherence and assumed effects*	Multi way analysis	One way analysis	One way analysis<br>*Sensitive to handling of drop-outs (inclusion of costs,assumed effect)*
**Sponsorship**	Government	Government	Government	Government

* Cost-effectiveness analysis; § Incremental cost-effectiveness ratio; ‡ Quality adjusted life year; † Cardio-vascular disease; ¶ Life years gained

Health economic evaluations of the prevention of cardiovascular diseases – Intervention target: **PHYSICAL INACTIVITY**; study 5

**Author**	**Di Loretto C**
**Year of publication**	**2005**
**Journal**<br>**(Journal category)**	**Diabetes Care**<br>*(Medicine)*
**Title**	**Make your diabetic patients walk**
**Objective**	To establish the impact of different amounts of increased energy expenditure on type 2 diabetes
**Intervention**	Weekly expenditure of >10 metabolic equivalents per hour (METS $\cdot$ h^{-1})
**Comparator**	Weekly energy expenditure<br>< 10 METS $\cdot$ h^{-1}
**Study setting**	Italy (Europe)
**Study design**	Retrospective observational analysis (of an randomized clinical trial)
**Type of evaluation**	Cost-consequence analysis
**Intervention target**	Physical inactivity
**Type of intervention**	Clinical prevention:<br>*Health education*<br>*(not described in detail)*
**Target group**<br>**(Gender, risk factor/co-morbidity, age)**	Male & female<br>Diabetes type 2<br>Age: 62 ± 0.7
**Analytic horizon**	2 years
**Discounting &**<br>**Base year**	Not discounted<br><br>Base year: 2000
**Perspective**	Not stated
**Benefit measurements**	Blood lipid level<br>Blood pressure<br>Blood glucose level<br>Weight loss<br>10-year CVD risk reduction

**Benefit results**	% change in10 year CVD-risk<br>(by weekly METS · h^{-1}):<br>0: + 0.1; 1-10: -0.3; 11-20: -2.6; 21-30: -3.7; 31-40: -4.8; >40: -4.3
**Costs included**	Direct costs: *No*<br>Resource consumption stated: *No*<br>Indirect costs: *No*<br>Future costs (saved):<br>*Medical costs, other healthcare*<br>Future costs (caused): *No*<br>Implementation costs: *No*
**Cost results**	Yearly $/ capita changes in drug costs (by weekly METS · h^{-1}):<br>0: + 393; 1-10: +206; 11-20: -196; 21-30: -593; 31-40: -660;<br>>40: -579
**Outcome ratio**	Not stated
**Methods to handle uncertainty**	No handling of uncertainty stated
**Sponsorship**	Not stated

* Cost-effectiveness analysis; § Incremental cost-effectiveness ratio; ‡ Quality adjusted life year; † Cardio-vascular disease; ¶ Life years gained

Health economic evaluations of the prevention of cardiovascular diseases – Intervention target: **VARIOUS**; studies 1-4

**Author**	**Lindholm L**	**Finkelstein EA**	**Baxter T**	**Baxter AP**
**Year of publication**	**1996**	**2002**	**1997**	**1997**
**Journal (Journal category)**	**Journal of Epidemiology and Community Health** *(Public Health)*	**Journal of Women's Health & Gender-Based Medicine** *(Public Health)*	**BMJ** *(Medicine)*	**Public Health** *(Public Health)*
**Title**	**Cost effectiveness and equity of a community based cardiovascular disease prevention programme in Norsjö, Sweden**	**Cost-effectiveness of a cardiovascular disease risk reduction program aimed at financially vulnerable women: The Massachusetts WISEWOMAN Project**	**A cost effective, community based heart health promotion project in England: prospective comparative study**	**The impact of heart health promotion on coronary heart disease lifestyle risk factors in schoolchildren: lessons learnt from a community-based project**
**Objective**	To evaluate the cost-effectiveness and equity of a community based cardiovascular disease prevention program	To evaluate two interventions aimed at cardiovascular risk reduction in financially vulnerable women	To determine whether a community based coronary heart disease health promotion project is cost-effective	To estimate the costs and effects of a school based heart health promotion program in modifying risk factors
**Intervention**	Health promotion activities undertaken in the community by adult educational associations, sports clubs, media, food retailers, companies, and local authorities combined with screening for risk factors and follow up advice	Enhanced lifestyle intervention (EI): Screening, one-on-one counseling session, further counseling, group intervention activities that focus on improving physical activity levels and nutrition	Several health promotion activities concerning behavior change, education, empowerment, medical therapy, and social environment change (details on www.bmj.com)	Health promotion at schools: peer-to-peer education, healthy eating days, no smoking policies, information leaflets and posters, special shows, curriculum activities
**Comparator**	No intervention	Minimum intervention (MI): Screening, one-on-one counceling	No intervention	No intervention
**Study setting**	Sweden (Europe)	USA (North America)	United Kingdom (Europe)	United Kingdom (Europe)
**Study design**	Observational trial and modeling	Observational trial and modeling	Observational trial and modeling	Prospective observational trial
**Type of evaluation**	CEA* (life years gained)	CEA* (life years gained)	CEA* (life years gained)	Cost-consequence analysis
**Intervention target**	Various	Various	Various	Various
**Type of intervention**	Health promotion: *Education (and various other)*	Clinical prevention: *Health education*	Health promotion: *Various*	Health Promotion: *Education*
**Target group (Gender, risk factor/co-morbidity,**	Male & female<br>Age: 30-60	Female<br>Age: ≥ 50	Male & female<br>Age: 18-64	Male & female<br>Age: 11-14

Health economic evaluations of the prevention of cardiovascular diseases – Intervention target: **VARIOUS**; studies 1-4

**Author**<br><br>**age)**	**Lindholm L**	**Finkelstein EA**	**Baxter T**	**Baxter AP**
**Analytic horizon**	15 years benefits (10 years costs)	Lifetime (model) 1-year (trial)	Lifetime (model) 4 years (trial)	3 years
**Discounting & Base year**	Costs: 5%<br>Benefits: 5%<br>Base year: 1992	Costs: No discounting reported<br>Benefits: 3%<br>Base year: 1996	Costs: 6%<br>Benefits: 6%<br>Base year: Not stated	Not discounted<br><br>Base year: Not stated
**Perspective**	Not stated	Not stated	Purchasing community<br>(Health authority)	Third party payer
**Benefit measurements**	Blood lipid level<br>Blood pressure<br>Avoided CVD[†]<br>Life years gained	10-year CVD[†] risk reduction<br>Life years gained	Blood lipid level<br>Blood pressure<br>Weight reduction<br>Behavior change<br>Life years gained	Change of behavior
**Benefit results**	Interv.: 4.5 cases of CVD[†] per year prevented	Incremental 10-year CVD[†] risk change (EI vs. MI) : -0.3%	Odds ratio compared to pre-interv.<br>- to drink low fat milk<br>Interv.: 2.58; Comp.: 1.81<br>- to smoke actively<br>Interv.: 0.83; Comp.: 1.1<br><br>➔ 3,581 life years gained	Odds ratio compared to pre-interv.<br>-to eat whole meal bread at age 11<br>Interv.: 1.3; Comp.: 0.84<br>- to drink low fat milk at age 11<br>Interv.: 2.01; Comp.: 2.28<br>- to exercise at age 11<br>Interv.: 1.76; Comp.: 0.41
**Costs included**	Direct costs: *Screening, administration, program costs*<br>Resource consumption stated: *No*<br>Indirect costs: *Participant time lost (human capital approach)*<br>Future costs (saved):<br>*Avoided CVD*[†]<br>Future costs (caused): *No*<br>Implementation costs: *No*	Direct costs: *Staff costs, material costs*<br>Resource consumption stated: *No*<br>Indirect costs: *No*<br>Future costs (saved): *No*<br>Future costs (caused): *No*<br>Implementation costs: *No*	Direct costs:<br>*Staff, consumable costs, meetings, events (preparing and executing), school expenditure*<br>Resource consumption stated: *Yes*<br>Indirect costs: *No*<br>Future costs (saved): *No*<br>Future costs (caused): *No*<br>Implementation costs: *No*	Direct costs: *activities and material*<br>Resource consumption stated: *No*<br>Indirect costs: *No*<br>Future costs (saved): *No*<br>Future costs (caused): *No*<br>Implementation costs: *No*
**Cost results**	Total: £ 363,000<br>Per year: £ 51,050<br>(costs saved not included)	EI: $ 603 per person<br>MI: $ 487 per person	Inter.: £ 108,774	Total: £ 16,350
**Outcome ratio**	Cost-saving - £ 14,900/LYG	Per 1% of 10-year CVD[†] risk reduction (ICER[§]): $ 637<br>Per LYG[¶] (ICER[§]): $ 4,966	£ 117 per life year gained	Not stated

Health economic evaluations of the prevention of cardiovascular diseases – Intervention target: **VARIOUS**; studies 1-4

**Author**	**Lindholm L**	**Finkelstein EA**	**Baxter T**	**Baxter AP**
**Methods to handle uncertainty**	Multi way analysis<br>Sensitive to included costs	No handling of uncertainty stated	One way analysis<br>*Only costs tested for uncertainty*	No handling of uncertainty stated
**Sponsorship**	Not stated	Government	Government	Government

* Cost-effectiveness analysis; § Incremental cost-effectiveness ratio; ‡ Quality adjusted life year; † Cardio-vascular disease; ¶ Life years gained

Health economic evaluations of the prevention of cardiovascular diseases – Intervention target: **VARIOUS**; studies 5-6

**Author**	**Murray CJL**	**Kumpusalo E**
**Year of publication**	**2003**	**1996**
**Journal**<br>**(Journal category)**	**The Lancet**<br>*(Medicine)*	**Health Promotion International**<br>*(Public Health)*
**Title**	**Effectiveness and costs of interventions to lower systolic blood pressure and cholesterol: a global and regional analysis on reduction of cardiovascular-disease risk**	**Finnish healthy village study: impact and outcomes of a low-cost local health promotion grogramme**
**Objective**	To estimate the cost-effectiveness of different interventions targeted at high-blood pressure and<br>-cholesterol levels	To study the impact and outcome of a low-cost local health promotion program in rural villages in Finland
**Intervention**	N1: Salt reduction through voluntary agreement with industry<br>N2: Population wide reduction in salt intake legislation<br>N3: Health education through mass media<br>N4: Combined intervention of N2 and N3<br>P1 and P2: Individual based hypertension treatment and education<br>P3 and P4: Individual treatment for high cholesterol concentrations and education<br>P6 to P9: Absolute risk approach for pharmacotherapy<br>C1 to C4: Combination of N4 with P6 to P9	- "Action group" was formed consistent of local men and women with prestige;<br>- In cooperation with the adult education institute, development of a program for spring and autumn including seminars with external lectures, study and sport groups, sport courses and a walking campaign<br>- Distribution of information through local radio and newspaper as well as by a program-books send to every household
**Comparator**	No intervention	No intervention<br>(for ethical reasons still some offer of activities, personal feedback and walking tests)
**Study setting**	Multi-national study	Finland (Europe)
**Study design**	Modeling	Prospective observational trial
**Type of evaluation**	Cost-utility analysis	Cost-consequence analysis
**Intervention target**	Various	Various
**Type of intervention**	Various health promotion & pharmacotherapy	Health promotion:<br>*Education*
**Target group**<br>**(Gender, risk factor/co-morbidity, age)**	Male & female<br><br>Age: Whole population	Male & female<br><br>Age: 20-64

Health economic evaluations of the prevention of cardiovascular diseases – Intervention target: **VARIOUS**; studies 5-6

**Author**	**Murray CJL**	**Kumpusalo E**	
**Analytic horizon**	Lifetime	3 years	
**Discounting & Base year**	Costs: 3%<br>Benefits: 3%<br>Base year: 2000	Not discounted<br><br>Base year: Not stated	
**Perspective**	Not stated	Not stated	
**Benefit measurements**	Disability adjusted life years (DALYs) averted	Blood lipid level<br>Blood pressure<br>Weight reduction	
**Benefit results**	DALYs (x 10^5) averted in EurA:<br>N1: 7; N2: 13; N3: 12; N4: 24; P1:73; P2: 82; P3: 65; P4: 69; P5: 110; P7: 95; P8:104; P9:114; C1: 91; C2: 99; C3: 106; C4: 116	% change in serum cholesterol (mmol /L):<br>Interv.: -10; Comp: -6<br>% change in mean plasma vitamin C concentration:<br>Interv.: +53; Comp.: +29	
**Costs included**	Direct costs: *Primary care visits, diagnostic test, medicines, side-effects, administration*<br>Resource consumption stated: *No*<br>Indirect costs: *No*<br>Future costs (saved): *No*<br>Future costs (caused): *No*<br>Implementation costs: *Office space, vehicles*	Direct costs: *Field costs of the survey, laboratory analysis, program costs*<br>Resource consumption stated: *No*<br>Indirect costs: *No*<br>Future costs (saved): *No*<br>Future costs (caused): *No*<br>Implementation costs: *No*	
**Cost results**	Costs ($ x 10^6) for EurA region:<br>N1: 297; N2: 297; N3: 202; N4: 499; P1:14,777; P2: 37,385; P3: 19,187; P4: 27,142; P5: 56,572; P7: 16,015; P8:22,226; P9:35,750; C1: 11,045; C2: 15,474; C3: 21,612; C4: 35,095	£ 40 per participant for evaluation<br>£ 750 extra costs per village/year	
**Outcome ratio**	Costs ($) / DALY averted in EurA:<br>N1: 440; N2: 230; N3: 160; N4: 210; P1: 2030; P2: 4540; P3: 2970; P4: 3930; P5: 5160; P7: 1680; P8:2150; P9: 3140; C1: 1210; C2: 1570; C3: 2030; C4: 3020	Not stated	
**Methods to handle uncertainty**	Multi way analysis	No handling of uncertainty stated	
**Sponsorship**	Not stated	Government	

* Cost-effectiveness analysis; § Incremental cost-effectiveness ratio; ‡ Quality adjusted life year; † Cardio-vascular disease; ¶ Life years gained
